# Endolysosomal targeting of a clinical chlorin photosensitiser for light-triggered delivery of nano-sized medicines

**DOI:** 10.1038/s41598-017-06109-y

**Published:** 2017-07-20

**Authors:** Elnaz Yaghini, Ruggero Dondi, Kunal M. Tewari, Marilena Loizidou, Ian M. Eggleston, Alexander J. MacRobert

**Affiliations:** 1 Division of Surgery and Interventional Science, University College London, Royal Free Campus, Rowland Hill Street, London, NW3 2PE UK; 2 Department of Pharmacy and Pharmacology, University of Bath, Bath, BA2 7AY UK

## Abstract

A major problem with many promising nano-sized biotherapeutics including macromolecules is that owing to their size they are subject to cellular uptake via endocytosis, and become entrapped and then degraded within endolysosomes, which can significantly impair their therapeutic efficacy. Photochemical internalisation (PCI) is a technique for inducing cytosolic release of the entrapped agents that harnesses sub-lethal photodynamic therapy (PDT) using a photosensitiser that localises in endolysosomal membranes. Using light to trigger reactive oxygen species-mediated rupture of the photosensitised endolysosomal membranes, the spatio-temporal selectivity of PCI then enables cytosolic release of the agents at the selected time after administration so that they can reach their intracellular targets. However, conventional photosensitisers used clinically for PDT are ineffective for photochemical internalisation owing to their sub-optimal intracellular localisation. In this work we demonstrate that such a photosensitiser, chlorin e_6_, can be repurposed for PCI by conjugating the chlorin to a cell penetrating peptide, using bioorthogonal ligation chemistry. The peptide conjugation enables targeting of endosomal membranes so that light-triggered cytosolic release of an entrapped nano-sized cytotoxin can be achieved with consequent improvement in cytotoxicity. The photoproperties of the chlorin moiety are also conserved, with comparable singlet oxygen quantum yields found to the free chlorin.

## Introduction

A major challenge encountered with many promising nano-sized biotherapeutics is that owing to their size they are subject to cellular uptake via endocytosis, and so become sequestered within endolysosomes. This can significantly reduce the therapeutic efficacy of such agents since they cannot reach their intended intracellular targets, and are also subject to endolysosomal degradation by proteolytic enzymes within lysosomes^1–3^. Photochemical internalisation (PCI) is a novel technology for enhancing the intracellular delivery and therapeutic efficacy of a range of bioactive agents that are prone to entrapment in endosomes and lysosomes^4–6^. The PCI technique uses visible light excitation in combination with a co-administered photosensitiser (PS) and is designed specifically to address the problem of sequestration of bioactive agents in endolysosomes, so it could also be applied not only to cancer but also non-cancerous lesions. PCI has proven effective in a wide range of experimental cancer models, including multidrug resistant cancer cells^6^, and has shown promising results in a clinical trial of head and neck cancer, using the chemotherapeutic agent bleomycin^7^. Although this entrapment is particularly problematic for nano-sized and macromolecular agents, some smaller drugs such as the chemotherapeutic agent doxorubicin may also become protonated and entrapped in acidic lysosomes owing to their weakly basic nature. This entrapment then limits transport and binding to nuclear DNA, which is the therapeutic target of doxorubicin. PCI is based on a spatio-temporal mechanism where a sub-lethal visible light dose is used to activate a photodynamic photosensitiser that localises in endolysosomal membranes, which then induces partial rupture of these intracellular organelles mediated by reactive oxygen species (ROS). This partial rupture enables the entrapped bioactive agents to escape and reach their intended target and exert their effect, but has been shown experimentally not to compromise the viability of the cells themselves^8^. For cytotoxic drugs therefore, PCI can enhance the killing using a lower drug dose thereby potentially alleviating toxic side-effects of the drug. PDT is already used clinically for a range of cancers and the light delivery technology can therefore be easily adapted for PCI^9, 10^. The endolysosomal release may also be triggered at a preselected time following administration of the drug using the light application in contrast to drug delivery systems which rely on lysosomal permeabilisation agents or chemical modification of the drug^3, 11^.

In order for PCI to function optimally and release the endolysomally entrapped agent, the photosensitiser used must possess a number of properties: firstly, it should localise in the same intracellular vesicles (lysosomes, endosomes) as the administered drug, i.e. they must be *lysosomotropic*. Secondly they should be amphiphilic in order to localise in the lipid bilayer membrane of the vesicle. If the photosensitiser instead localises within the inner aqueous compartment of the vesicles they are ineffective for PCI, as shown by comparing the PCI properties of di- and tetrasulfonated derivatives of tetraphenylporphine. Berg and colleagues found that only the amphiphilic disulfonated derivative was effective for PCI, whereas the more water soluble and less amphiphilic symmetrical tetrasulfonated derivative was ineffective, since it partitions to the aqueous compartment^12^. The same considerations have prompted the development of a disulfonated chlorin photosensitiser for clinical PCI^7^. The endolysosomal localisation of the disulfonated derivative is accounted for by uptake via adsorptive endocytosis. In this process, the hydrophobic part of the macrocycle is embedded in the cell membrane whereas the sulfonate groups are negatively charged and reside at the membrane-aqueous medium interface. Since red light penetrates more deeply into tissue owing to lower endogenous chromophore absorption (e.g. haemoglobin), the photosensitiser should ideally possess strong red or near-infrared (NIR) absorption.

Conventional PDT photosensitisers that are used clinically are unsuitable for PCI despite good red/NIR absorption and high quantum yields for ROS (e.g. singlet oxygen) generation since they partition non-selectively to other cellular organelles (e.g. mitochondria, Golgi apparatus, endoplasmic reticulum). The challenge therefore is how to harness the good optical and photophysical properties and low toxicity of conventional photosensitisers for PCI. In this context, there has been considerable interest in the development of peptide and protein-targeted photosensitisers to provide improved pharmacokinetic properties, solubility and tissue specificity in PDT. Conjugation of photosensitisers to antibodies and a range of synthetic peptides has been explored and has been found to provide significant enhancement in efficiency and selectivity of cellular uptake of photosensitisers for PDT in a range of cancer models^13–16^. For example Bisland *et al*.^17^ demonstrated enhanced PDT efficacy using a nucleus-directed linear peptide conjugated with chlorin e_6._ Cell-penetrating peptides (CPPs) have been widely investigated as delivery systems for targeted drug delivery^18–20^. CPPs typically consist of 8–30 amino acid residues and can translocate diverse molecular cargoes across biological membranes and transport, either covalently or non-covalently attached, which would otherwise be poorly internalised. This provides some interesting opportunities for light-based therapies such as PDT, and the conjugation of tetrapyrrole-based photosensitisers to CPPs has already been shown to enhance photosensitiser delivery for both PDT of cancer^21–23^ and in antimicrobial PDT applications^24, 25^.

We have sought to extend this principle by hypothesizing that attachment of an otherwise conventional photosensitiser to an appropriate CPP peptide sequence should transform it into an amphiphilic compound that is both water-soluble and amenable to cellular uptake by adsorptive endocytosis^26^. Most Arg-rich CPPs (e.g. Tat (48–57)) enter cells via endocytic processes, especially at low micromolar to sub-micromolar concentrations relevant for their use as *in vivo* delivery vectors^27^. A CPP-photosensitiser conjugate should therefore be suitable for PCI, since it should be able to localise in the lipid bilayer of the endosomal membranes, to deliver selective oxidative damage, with the hydrophilic protonated peptide residing at the membrane-aqueous interface and the aromatic photosensitiser macrocycle in the lipid bilayer. We have demonstrated that the conjugation of a porphyrin derivative to the Tat (48–57) sequence and other cationic CPPs is indeed an effective way of generating a novel water-soluble, amphiphilic photosensitiser suitable for light-triggered drug delivery by PCI^26, 28^. Since PCI involves light-triggered rupture of the endolysosomes which will result in intracellular dispersal into the cytosol of both the entrapped agent and the photosensitiser, this process can be visualised using the intrinsic fluorescence of the dispersed photosensitiser. Pellois and colleagues^29^ and Okazaki and colleagues^30^ have documented similar approaches to light-triggered release using CPP-targeting of non-porphyrin compounds, and in a related study we have demonstrated that visible light-triggered intracellular dispersal of lysosomally entrapped CPP-labelled photoluminescent quantum dots can be effected using PCI with an amphiphilic disulfonated phthalocyanine photosensitiser concomitant with dispersal of the photosensitiser fluorescence^31^. These results further highlight the potential of CPP-targeting to exploit photosensitisers with a selected spectroscopic profile for PCI.

**Figure 1:**
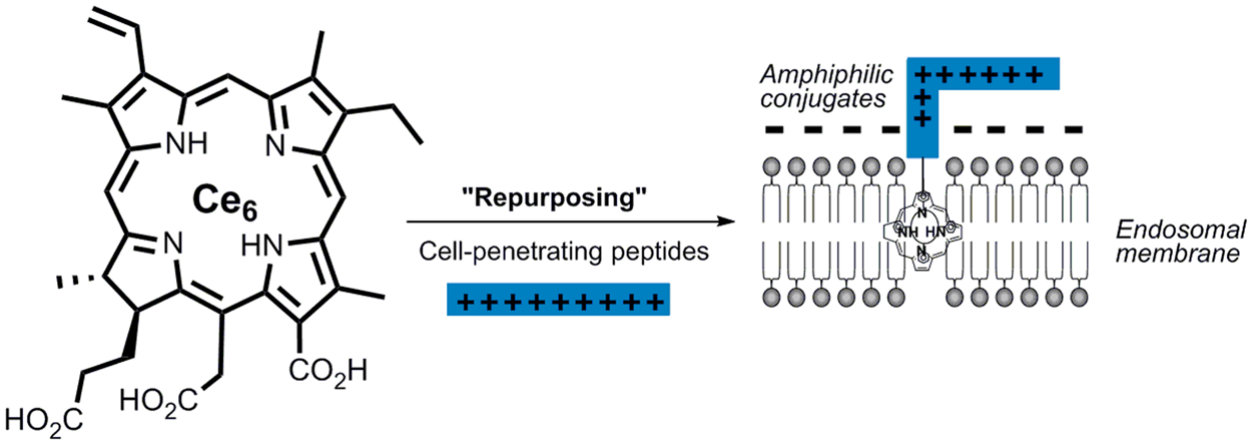
Concept of interfacial lipid membrane localisation of a chlorin e_6_ (Ce_6_) cell-penetrating peptide conjugate for light-triggered endosomal membrane rupture following oxidation of membrane components (eg unsaturated lipids) by reactive oxygen species.

A key innovation in the development of effective peptide-targeted photosensitisers has been the application of bioorthogonal ligation techniques^32^ which enable efficient regioselective attachment of photosensitisers to unprotected, multifunctional peptides and proteins in solution. This now provides the means for the repurposing of photosensitisers with real clinical potential. Many clinical PDT photosensitisers such as chlorin e_6_ or mTHPC are administered using a delivery system to improve water solubility consisting of an emulsion or liposomes (eg Verteporfin, which is formulated in liposomes as Visudyne)^9, 10^. The clinical formulation of chlorin e_6_ (Photolon) contains polyvinylprrrolidone (PVP) which is a synthetic neutral polymer used to aid drug dissolution and disaggregation^33, 34^. There are several recent studies investigating new routes to improve chlorin e_6_ delivery using other solubilising agents^35–37^ and nanoparticles^38, 39^ which underscore the interest in improving the pharmacokinetic properties of this photosensitiser. In this study we therefore selected chlorin e_6_ as the photosensitiser since it is already used clinically for PDT and has good red wavelength absorption properties, unlike porphyrin photosensitisers. Moreover, chlorin e_6_ exhibits poor lysosomal localisation^40^ therefore it is an ideal candidate for repurposing using our CPP-targeting approach, with the concept depicted in Fig. 1. The aim of this study was thus to utilise bioorthogonal ligation chemistry to target chlorin e_6_ for PCI by regioselective attachment to a typical cationic CPP, namely Tat (48–57), thereby providing enhanced cellular uptake and controlled intracellular localisation.

## Results and Discussion

### Synthesis of Tat-conjugated chlorin derivatives

**Figure 2:**
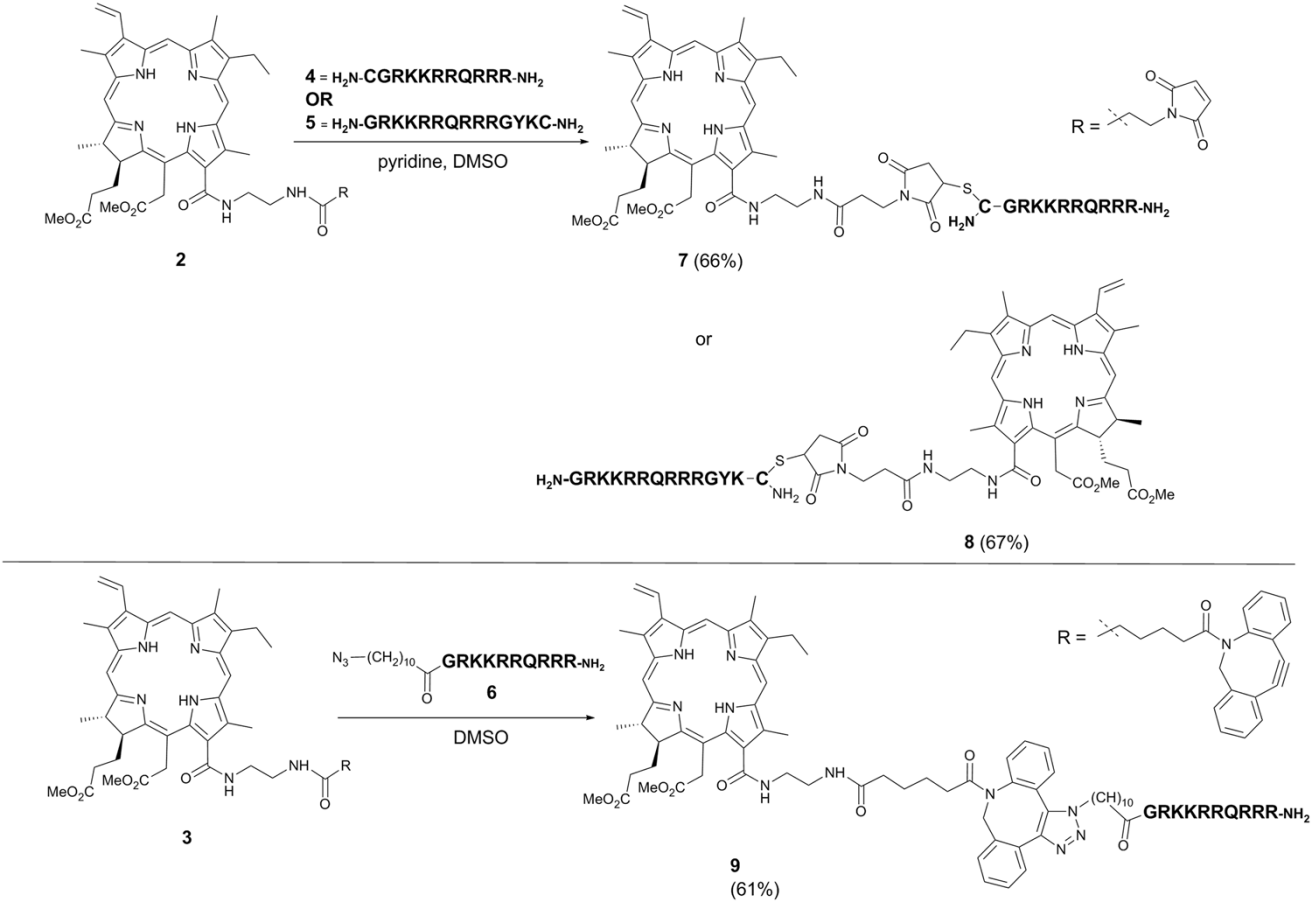
Synthesis of chlorin-Tat conjugates **7–9**.

Ligatable 13^1^-linked derivatives of chlorin e_6_, **2** and **3**, were obtained by regioselective functionalisation of methyl pheophorbide a with an ethylenediamine spacer^41, 42^, followed by acylation with the appropriate carboxylic acid (see Supplementary Information). Tat peptide derivatives **4** and **5** containing a cysteine residue at the N- or C-terminus were synthesised by standard 9-fluorenylmethoxycarbonyl (Fmoc) solid phase peptide synthesis on Rink amide resin, with peptide **6** being obtained by acylation of the N-terminus of the Tat (48–57) sequence with 11-azidoundecanoic acid^28^. Conjugation of chlorins **1** and **2** to the Tat derivatives **4–6** were carried out as recently described (see Scheme 2)^28^. Thiol-maleimide coupling with the maleoyl-chlorin derivative **2** and the N- or C-terminally functionalised peptides **4** and **5**, proceeded efficiently in DMSO to give the desired conjugates **7** and **8** in 66% and 67% yields respectively. Strain-promoted azide-alkyne cycloaddition (SPAAC) between the cyclooctyne-bearing chlorin derivative **3** and peptide **6** in DMSO gave conjugate **9** as a mixture of triazole regioisomers in 61% yield. The reaction scheme is shown in Fig. 2.

### Photophysical studies

Spectral absorbance studies of the conjugates showed the peak absorption of the Soret band and the longest wavelength red Q band absorption peak were observed at very similar wavelengths 404 +/− 1 nm and 662 +/− 1 nm respectively in 0.1% trifluoroacetic acid (see Supplementary Information). For the determination of the singlet oxygen quantum yield (Φ_Δ_), deuterated methanol (CD_3_OD) was used since singlet oxygen has a relatively long lifetime in deuterated solvents. To confirm that the methanolic solutions of the conjugates and chlorin e_6_ at the concentrations studied were monomeric, absorption spectra were recorded as a function of concentration. This was necessary because aggregated photosensitisers generally exhibit lower extinction coefficients, and weak fluorescence and low singlet oxygen quantum yields. The peak absorbance of the Soret band, at 398 nm for the conjugates and 399 nm for chlorin e_6_, was found to be linear versus concentration up to 8 μM confirming that the chlorin conjugates were present in a monomeric form; the red Q band peak is at 661 nm for all compounds. The singlet oxygen quantum yields in deuterated methanol were measured using a standard reference compound, Rose Bengal (Φ_Δ_ = 0.76)^43^ using matched reference solution absorbances at the laser wavelength of 532 nm. Using standard zero-point intercept analysis of the singlet oxygen decays, the quantum yield of chlorin e_6_ was calculated as 0.67, which is in good agreement with the literature, as reviewed by Redmond *et al*.^43^. Morjzisova *et al*. reported the quantum yields of several chlorins in dimethylformamide and measured a value of 0.63 for chlorin e_6_, and observed a similar value for another clinical chlorin PDT photosensitiser, mTHPC (0.68) and disulfonated tetraphenyl chlorin (0.66)^44^.

The singlet oxygen quantum yields of the N- and C-functionalised conjugates **7** and **8** were measured as 0.62 and 0.69 respectively; the difference is not statistically significant given the estimated experimental error of +/− 0.06. Addition of azide ions, a powerful singlet oxygen quencher, at 5 mM strongly quenched the emission (Supplementary Information). The similarity found between the singlet quantum yields for the conjugates and that of chlorin e_6_ shows that attachment of the peptide does not adversely affect the singlet oxygen yield, and also shows that switching the location of the photosensitiser within the peptide backbone does not have a significant effect on the efficiency of singlet oxygen production. The importance of the role of singlet oxygen in the PCI mechanism has recently been examined by Okazaki and colleagues^30^ who demonstrated a significant correlation between the rate of endosomal rupture and the relative quantum yields of photogenerated singlet oxygen.

### Uptake and subcellular localisation

**Figure 3:**
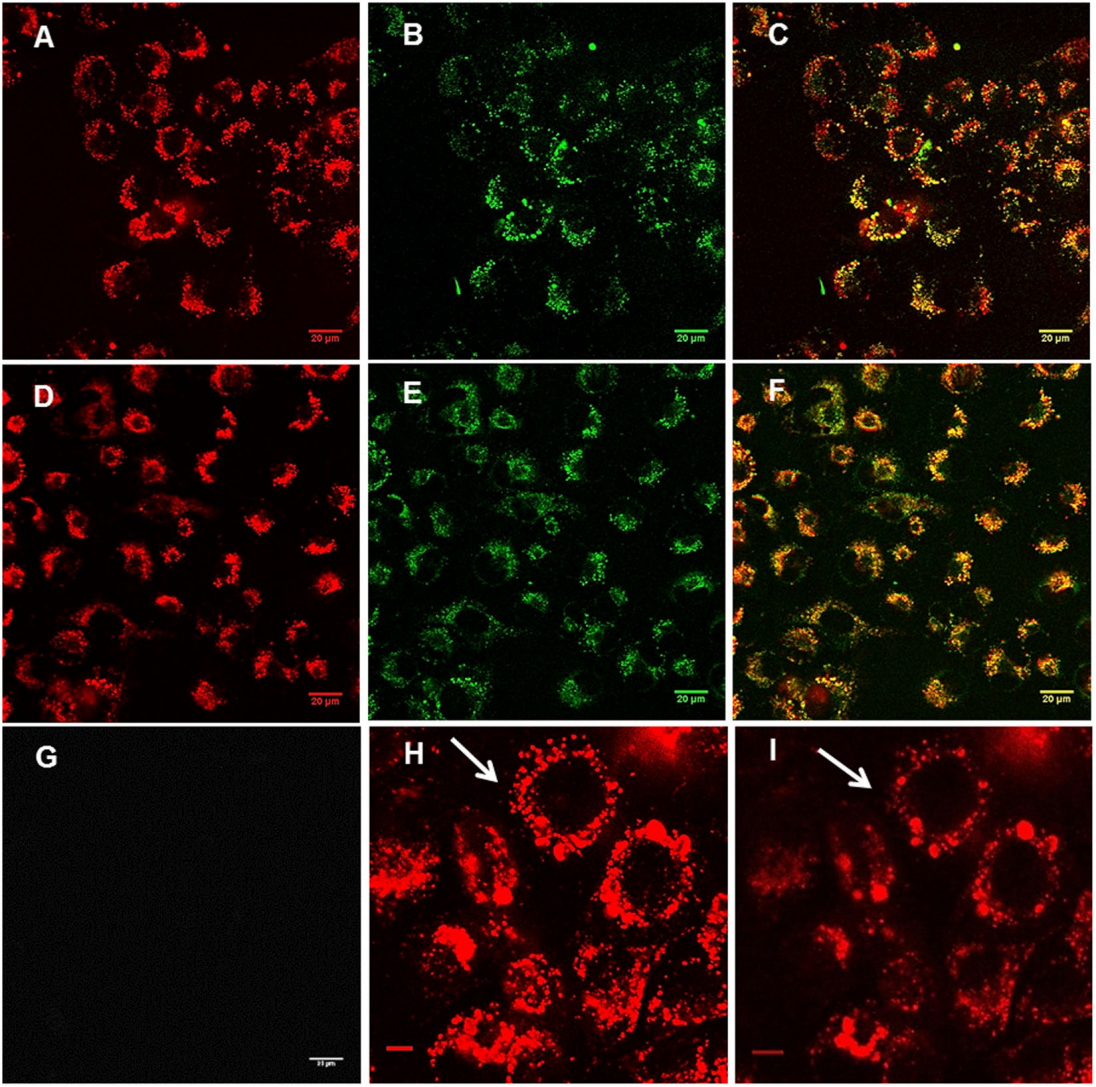
Cellular uptake and colocalisation of the conjugates with LysoTracker Green in MC28 cells using laser scanning confocal microscopy. Cells were incubated with the conjugates for 24 h. LysoTracker Green was applied to cells 30 minutes before imaging. (**A**) Conjugate **7** alone (red), (**B**) LysoTracker Green (green), (**C**) merged (**A** and **B)**. Scale bar: 20 μm; (**D**) conjugate **8** alone (red), (**E**) LysoTracker Green (green), (**F**) merged (**D** and **E**). Scale bar: 20 μm; (**G**) MC28 cells following incubation with unconjugated chlorin e_6_. Scale bar: 20 µm; (**H and I**) Cellular fluorescence of conjugate **7** (**H**) measured initially, and (**I**) after prolonged on-stage laser illumination at 405 nm. Arrows highlight areas where fluorescent vesicles initially observed are absent following laser illumination. Scale bar: 5 µm.

**Figure 4:**
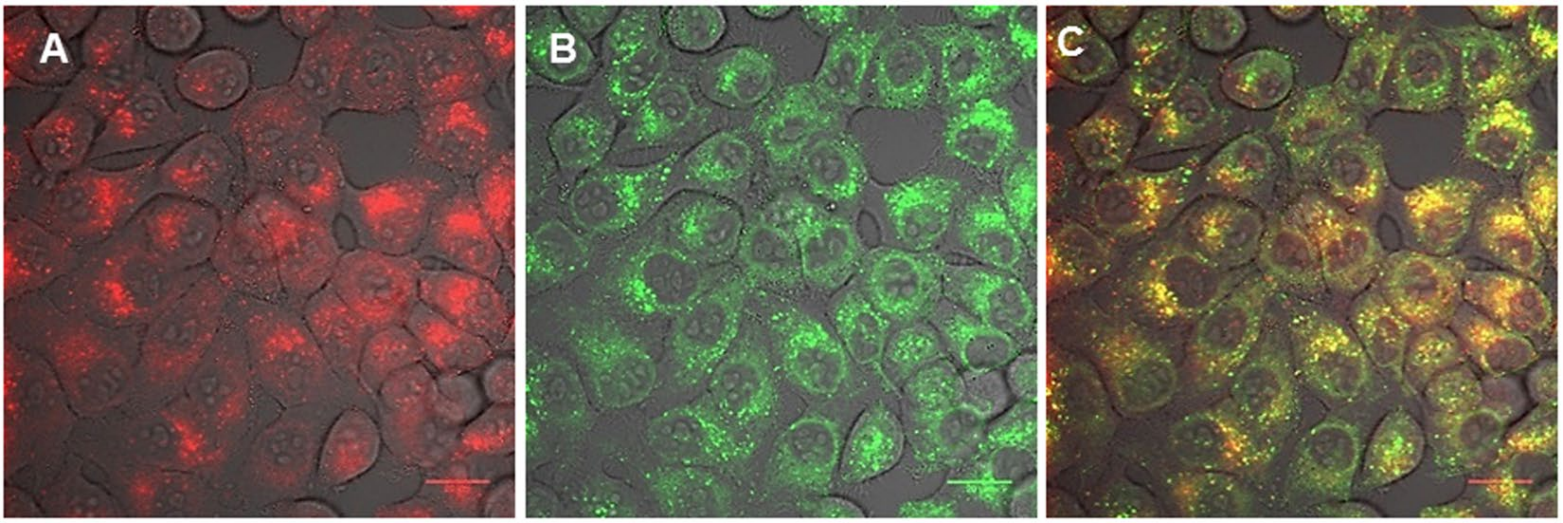
Cellular uptake and colocalisation of conjugate **8** with LysoTracker Green in KB cells using laser scanning confocal microscopy. Cells were incubated with the conjugate for 24 h. LysoTracker Green was applied to cells 30 min before imaging. (**A**) Conjugate alone (red), (**B**) LysoTracker Green (green), (**C**) merged (**A and B**). Scale bar: 20 μm.

Cellular uptake of the conjugates was examined by confocal microscopic imaging of the photosensitiser fluorescence in MC28 cells. Both of the N-terminally linked conjugates 7 and 9 showed a punctate subcellular localisation (Fig. 3). Colocalisation of the photosensitiser fluorescence with lysosomes was demonstrated using the lysosomal marker, Lysotracker Green, as visualised by the yellow colour in the merged images. Figure 4 shows the same pattern of colocalisation was observed for the C- terminally linked conjugate **8** in the KB cell line. The good subcellular colocalisation observed between the photosensitiser fluorescence of **7–9** and the lysosomes therefore indicates that they are potentially suitable photosensitisers for PCI applications.

Using the same concentration of free chlorin e_6_ and image intensity scale, much lower fluorescence was observed (Fig. 3G), which shows that conjugation with the CPP significantly enhances the cellular uptake of the photosensitiser. The lower uptake of the free chlorin is consistent with its lower PDT efficacy compared to the conjugates, as discussed below. Similarly, Bastien *et al*.^40^ found that uptake in human pharyngeal FaDu carcinoma cells of free chlorin e_6_ was an order of magnitude lower compared to delivery using dendrimer conjugates of chlorin e_6_. Figure 3H and I show sequential images of the effect of prolonged on-stage illumination on the intracellular fluorescence of conjugate 7 using the confocal microscope 405 nm laser. Acquisition of image H used an exposure time of only 1 s, which did not result in detectable perturbation of the nascent fluorescence distribution. However after a further 30 s of exposure to the laser, a repeat image scan (with a 1 s exposure) showed that the intracellular fluorescence had largely dispersed (see arrow) and that only a few of the original fluorescent vesicles were evident (Fig. 3I). This dispersal of fluorescence is consistent with the PCI mechanism whereby the endolysosomal membranes, which harbour the photosensitiser, are disrupted following illumination^8, 30, 31^. The lipid bilayer contains several substrates such as unsaturated lipids and cholesterol, which stabilise the membrane structure, that are susceptible to photooxidative degradation induced by the reactive oxygen species such as singlet oxygen which are formed after exciation of the photosensitser. This oxidative damage then induces structural breakdown of the membrane resulting in photosensitiser dispersal. However photochemically-induced disruption of endolysosomal membranes by itself has been shown to be a sub-lethal process^8^ and it is the release of the toxin within the endolysosomal compartment that is responsible for the lethal effect of PCI using cytotoxins. No evidence of nuclear localisation was observed for **7–9** in keeping with other studies by Vicente and colleagues and our own previous work^22, 28^. In contrast there have been reports of fluorescently-labelled nanoparticles conjugated with the Tat peptide^38^ which do show uptake in the nucleus. However this is probably accounted for by conjugation of multiple Tat moieties to the surface which then assumes a strong cationic charge favouring nuclear localisation.

### Phototoxicity studies

**Figure 5:**
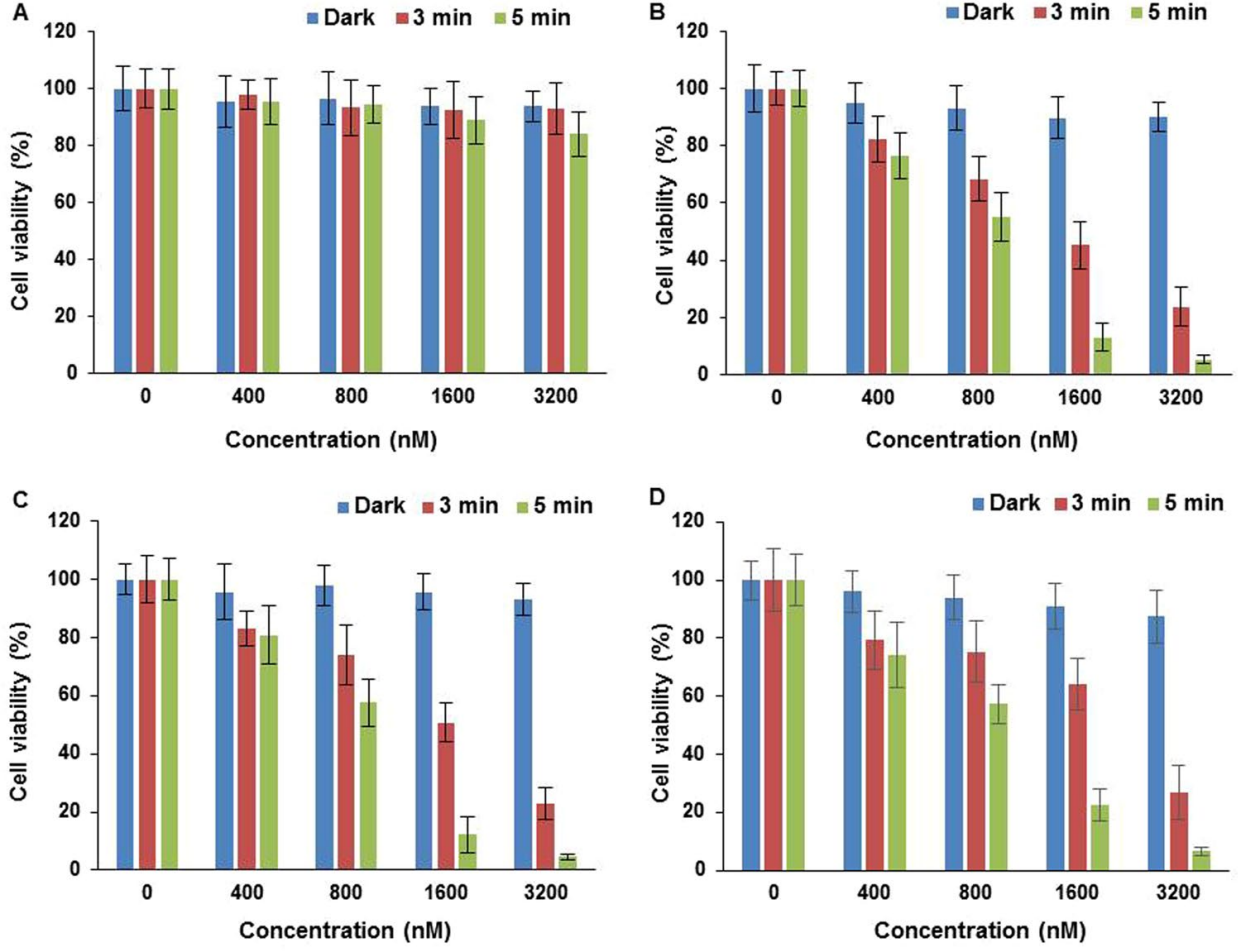
PDT effect of chlorin e_6_ without Tat-peptide conjugation (**A**), **7** (**B**), **8** (**C**) and 9 (**D**) in MC28 cells. Cells were incubated with the compounds at various concentrations and were illuminated for either 3 or 5 minutes. MTT assay was carried out 48 h after light exposure. Data are presented as mean value ± standard deviation (SD) of three independent experiments.

**Figure 6:**
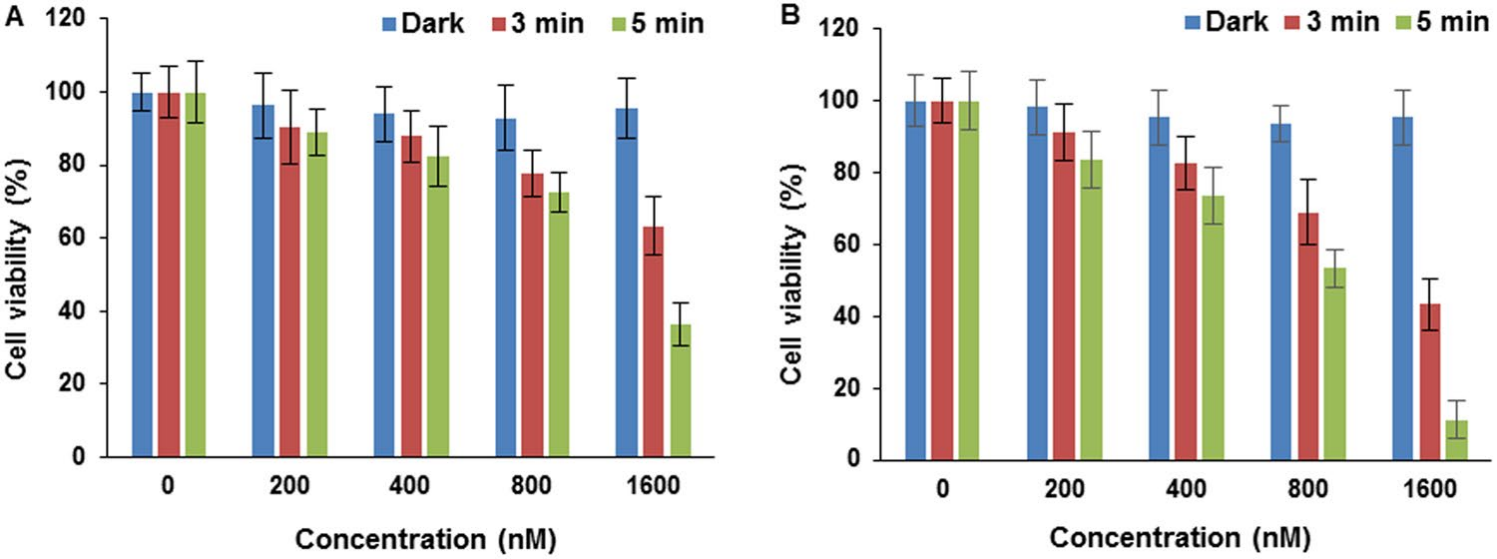
PDT effect of **7** (**A**) and **8** (**B**) KB cells. Cells were incubated with the conjugates at various concentrations and were illuminated for either 3 or 5 minutes. MTT assay was carried out 48 h after light exposure. Data are presented as mean value ± standard deviation (SD) of three independent experiments.

The photoxicities of the N- and C-terminally linked maleoyl conjugates **7** and 8, and the C-terminally linked triazole conjugate 9 were examined in MC28 cells, as shown in Fig. 5, as a function of dose and illumination time. Significant phototoxicity was observed with all compounds and was enhanced at higher photosensitiser concentration and light doses. At the highest dose used of 3.2 µM, greater than 90% loss of viability was observed for all conjugates using a 5 min illumination, and the phototoxicity profiles appear to be very similar for the conjugates. In contrast however, unconjugated chlorin e_6_ was far less potent for PDT with less than 20% loss of viability observed at the same light and drug dose, as shown in Fig. 5A. Figure 6 shows the results for **7** and **8** in the KB cell line, also demonstrating progressive loss of viability with higher light and drug doses. Control experiments in the dark for the conjugates in both cell lines showed that there was no chemical toxicity at concentrations significantly greater than those typically employed for the PCI experiments (see below). From Fig. 3, which shows the relative intracellular photosensitiser fluorescence, the weaker phototoxicity of chlorin e_6_ is accounted for by its less efficient cellular uptake^17, 40^. In conjugate 7, the chlorin is linked to the N-terminus of Tat (48–57) via cysteine, and in **8** the chlorin is linked to the C-terminus of Tat (48–57), again via cysteine, but as part of a GYKC peptide extension. Thus while the linker between the peptide and the chlorin is the same for both **7** and 8, the latter bears two more positive charges at physiological pH. In conjugate 9, the N-terminus of Tat (48–57) is linked to the chlorin via an aliphatic spacer and a triazole linkage (using SPAAC ligation which removes one positive charge compared to the native CPP sequence.

Notwithstanding the changes in the peptide carrier that are inherent in switching the location of the chlorin photosensitiser, and also alteration of the linker, our results show that there are no significant differences in photodynamic efficacy between conjugates **7** and **9** (N-terminally linked) and **8** (C-terminally linked). All three conjugates are based upon selective 13^1^-functionalisation of the chlorin e_6_ structure, and it is relevant to note that previous studies by Smith and colleagues have found that chlorin e_6_ derivatives which are linked to amino acids via the 13^1^ carboxylic acid group show superior phototoxicities compared to isomeric derivatives obtained from conjugation to either of the other two acid functions^42^.

### Photochemical Internalisation studies (PCI)

**Figure 7:**
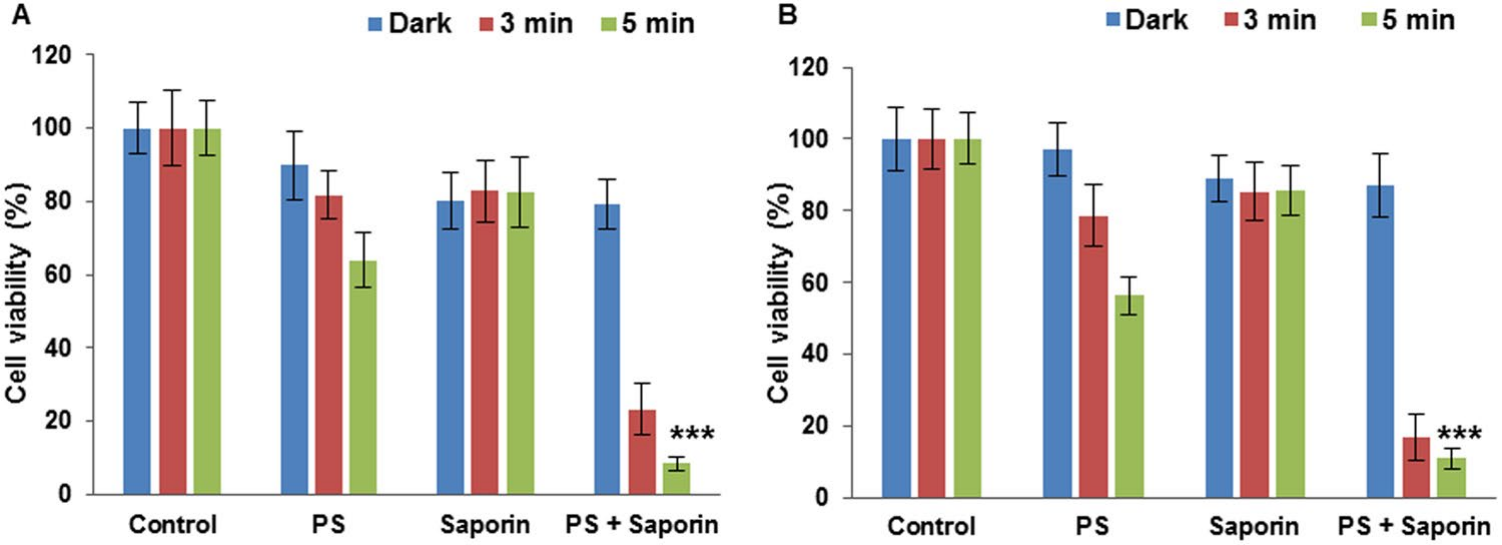
Light-induced cytotoxic response for **7** and **8** in KB cells, showing PDT (without saporin) and PCI (with saporin) effects. Cells were incubated with photosensitiser (550 nM) with or without saporin (20 nM) for 18 h. Illumination was carried out for 3 and 5 minutes, and the MTT assay was carried 96 h after illumination. Data are presented as mean value ± standard deviation (SD) of three independent experiments.

Conjugates **7** and **8** were tested for the light-triggered PCI enhancement in the cytotoxicity of saporin, which is a 30 kDa ribosome inactivating protein and has been used as a model nano-sized agent for PCI studies^4^. Owing to its size (c. 2 nm in diameter) saporin is prone to entrapment and degradation within lysosomes following endocytosis, thus severely restricting its cytotoxicity when administered directly^45^. The KB human oral epidermoid carcinoma cell line was used for these studies since head and neck cancer is one of the main clinical targets for PCI^7^. Treatment with saporin alone at 20 nM resulted in a small reduction in viability at <20%, as shown in Fig. 7. To test for PCI, low dose PDT treatment for both **7** and **8** was applied using lower conjugate doses so that most of the cells remained viable.

PCI treatment for both **7** and **8** resulted in significant reductions in viability of ~ 90% using 5 minutes illumination and 20 nM saporin (P < 0.0001). Thus PCI resulted in a reduction of viability compared to PDT alone by factors of 7.5 (conjugate **7**) and 5.2 (conjugate **8**) owing to the enhanced cytotoxicity exerted by the saporin following cytosolic release.

These PCI results compare favourably with those obtained with analogous N- and C-terminal Tat conjugates bearing a highly hydrophobic tetraphenylporphine moiety^28^ and the disulfonated tetraphenylporphine derivative^46^. Comparing the alpha values calculated using equation 1 (Methods) to ascertain whether a synergistic effect is observed, alpha values of 6.2 and 4.4 are obtained for **7** and 8, which is consistent with a synergistic effect (ie alpha greater than unity) attributable to PCI. If the effect of the combination treatment were merely additive, a value of unity would be observed. These data show that for PCI the effect of combining each conjugate with saporin is synergistic rather than additive and confirm that the site of functionalisation within the peptide is not critical to the PCI efficacy.

The rationale for selecting chlorin e_6_ as the photosensitiser for this study on PCI was based on its favourable photosensitising properties but also on its poor lysosomal localisation^40^ and bioavailability. The cellular uptake and membrane transport of chlorin e_6_ has been studied in detail in relation to its acid-base properties. The structure of chlorin e_6_ contains three adjacent carboxylic acid functions with pKa7.9–8.3^47^which are thus only partially ionised at physiological pH leading to poor aqueous solubility, which complicates systemic administration. At lower pH, as is typical for the microenvironment of solid tumours, increased protonation of the carboxyl groups leads to a change in lipophilicity^48^. In this case for a lipophilic compound, uptake via diffusion across cell membranes will predominate. Poor lysosomal localisation of chlorin e_6_ has indeed been observed which is accounted for by uptake via diffusion across the cell membrane^40^. This is in contrast to sulfonated tetrapyrrole derivatives which are effective for PCI, wherein the sulfonate groups are fully ionised at physiological pH (and also at the lower lysosomal pH), promoting water solubility and cellular uptake by adsorptive endocytosis^49^. For the CPP-chlorin conjugates **7–9** studied, the peptide moieties are highly hydrophilic, being protonated at physiological and lysosomal pHs, whereas the chlorin unit with both 17^3^- and 15^2^ -acid functions masked as esters is relatively hydrophobic. Conjugates **7–9** thus have the desired amphiphilic properties for PCI with a lipophilic photosensitiser moiety able to be embedded within the lysosomal membrane lipid bilayer and the hydrophilic cationic peptide component residing at the negatively charged membrane surface. The chlorin moiety would remain uncharged and relatively lipophilic in lysosomes even after hydrolysis of the esters groups owing to the pKa values ~ **8** of the carboxylic functions. The insertion of an additional relatively hydrophobic spacer unit between the chlorin and peptide moieties, especially in the case of **9** may be a further advantage in this respect as it potentially enables deeper penetration of the chlorin macrocycle into the membrane lipid bilayer for more effective oxidative damage to the membrane^44^.

## Conclusions

There has been considerable interest in developing new delivery systems for the clinical photosensitiser chlorin e_6_ to improve its bioavailability for photodynamic therapy, since it induces efficient generation of reactive oxygen species and has good red wavelength absorption properties, thereby enabling a deeper therapeutic effect^35–39^. In this work we have attempted to develop a different approach to exploiting the photosensitising properties of chlorin e_6_ by adapting it instead for light-triggered drug delivery via photochemical internalisation to address the problem of endolysosmal sequestration^4–6^. In this study we have demonstrated that by attaching a typical CPP to chlorin e_6_ using regiospecific bioorthogonal ligation chemistry, we can not only improve the cellular uptake but also achieve endolysosomal targeting of the photosensitiser. PCI treatment using a cytotoxic ribosome inactivating protein as a model nano-sized therapeutic agent was then successfully demonstrated. We also found that the singlet oxygen quantum yields and the cellular PCI efficiencies of the repurposed chlorin e_6_ derivatives were insensitive to which peptide terminus was employed for coupling to the native photosensitiser. Moreover, the PDT properties were improved as a result of the improved bioavailability and water solubility imparted by the peptide component. While PDT is an established clinical modality used solely for eradication of malignant or diseased lesions, PCI is a more versatile technique that can be used to enhance delivery of drugs for a range of purposes. Potentially however, PCI could replace PDT for treatment of malignant tumours in many cases since PCI can use established chemotherapeutic agents which should help PCI gain acceptance amongst clinical practitioners, and the fibre-optic laser light delivery technology needed for PCI can be based on what is already available for PDT. In summary, we have shown that amphiphilic chlorin peptide conjugates can be prepared using versatile bioorthogonal ligation chemistry under very mild conditions to generate novel derivatives which are effective for both PDT and PCI. This opens the way to apply this strategy further using CPPs with inherent tumour-homing properties^50^ and other photosensitisers that absorb in the NIR to provide tools for PCI with dual light and carrier-based selectivity and maximum clinical potential.

## Methods

### Materials

Chemical reagents were purchased from Sigma-Aldrich, Fluka, Acros, Novabiochem, and Bachem. Peptide grade dimethylformamide (DMF) was purchased from Rathburn Chemicals. Anhydrous dichloromethane (DCM) was obtained by distillation over calcium hydride. Analytical TLC was performed using silica gel 60 F_254_ pre-coated on aluminium sheets (Merck). Column chromatography was performed on silica gel 60 (35–70 micron) from Sigma-Aldrich.

### Characterizations

UV spectra were recorded on a Perkin-Elmer Lambda 19 uv/vis spectrophotometer. Fluorescence spectra were recorded on a Cary Eclipse fluorimeter. ^1^H and ^13^C NMR were recorded using a Varian Mercury-VX spectrometer at 400 MHz (^1^H) and 100 MHz (^13^C) or a Bruker Avance III 500 at 500 MHz (^1^H) and 125 MHz (^13^C). Chemical shift values are given in ppm (δ). J values are given in Hz. Analytical RP-HPLC was performed on a Dionex Ultimate 3000 system (Dionex, UK), with a VWD-3400 variable wavelength detector, and a RF-2000 fluorescence detector. Analyses were performed at 35 ± 0.1 °C on a Gemini 5 μ C18 110 A column, (150 × 4.6 mm - Phenomenex, UK), equipped with a Security Guard C18 (ODS) 4 × 3.0 mm ID guard column (Phenomenex, UK), at a flow rate of 1 mL/min. Mobile phase A was 0.1% aq. TFA, mobile phase B was 0.1% TFA in MeCN. (Gradient: 0.0–10.0 min 0–95% B, 10.0–20.0 min 95% B, 20.0–20.1 min at 95–5% B, 20.1–23.0 min 5% B). Preparative RP-HPLC was performed on a Dionex HPLC system equipped with a Phenomenex Gemini 5 μ C18 (250 × 10w mm) column at a flow rate of 2.5 mL/min. High resolution mass spectrometry was performed using a Bruker MicroTOF autospec ESI mass spectrometer.

### Synthesis of ligatable peptides (4)-(6)

Cysteine-containing Tat-peptides **4** and **5** were synthesised by Fmoc solid phase peptide synthesis on Rink amide resin as described previously^28^. Peptide **6** was obtained by N-terminal derivatisation of the resin-bound Tat (48–57) sequence with 11-azidoundecanoic acid using HATU activation^28^.

### Synthesis of Chlorin e_6_-Tat conjugates (7)-(9)

#### Conjugate (7) – N-terminal thiol maleimide ligation

A solution of Cys-Tat peptide 4 (35.0 mg, 17.8 mmol) and 2 (45 mg, 56.7 mmol) in DMSO (2.5 mL) was treated with pyridine (310 µL) and stirred at room temperature overnight, shielded from light. The mixture was diluted with 1.0% aq TFA and directly purified by semi-preparative HPLC (see Characterizations). The purified conjugate was freeze-dried to give **7** as a dark green solid (38.6 mg, 66%). HPLC t_R_: 8.82 min; UV-vis (0.1% aq. TFA), nm (%): 404 (100), 498 (6.5), 663 (22.6); fluorescence λ_max._ (0.1% aq. TFA, λ_exc_ = 405 nm) 652 n m; HRMS [found (ESI+): 773.09080 [M + 3 H]^3+^, calcd. for C_109_H_160_N_39_O_17_S: 773.0853].

#### Conjugate (8) – C-terminal thiol maleimide ligation

A solution of Tat-GYKC peptide **5** (78.5 mg, 26.3 μmol) and **2** (43 mg, 55.1 μmol) in DMSO (20 mL) was treated with pyridine (600 µL) and stirred at room temperature overnight, shielded from light. The mixture was diluted with Et_2_O and TFA (650 µL) was added dropwise. A mixture of conjugated and unconjugated peptide precipitated out, which was collected by centrifugation and dissolved in 1.0% aq. TFA (20 mL). This solution was then purified by semi-preparative HPLC (see General Information). The purified conjugate was freeze-dried to give **8** as a dark green solid (67.1 mg, 67%). HPLC t_R_: 6.44 min; UV-vis (0.1% aq. TFA), nm (%): 403 (100), 500 (19.7), 531 (11.3), 607 (12.9), 662 (43.1); fluorescence λ_max._ (0.1% aq. TFA, λ_exc._ = 405 nm) 653 nm; HRMS [found (ESI+): 888.8355 [M + 3 H]^3+^, calcd. for C_120_H_193_N_44_O_24_S 888.8313].

#### Conjugate (9) – N-terminal SPAAC ligation

A solution of azidoundecanoyl-Tat peptide **6** (7.8 mg, 3.1 μmol) and **3** (7.0 mg, 7.1 μmol) in DMSO (750 µL) was treated with pyridine (3 µL) and stirred at room temperature overnight, shielded from light. The mixture was diluted with 1.0% aq. TFA and directly purified by semi-preparative HPLC (see General Information). The purified conjugate was freeze-dried to give **9** as a dark green solid (4.0 mg, 61%). HPLC t_R_: 7.24 min; UV-vis (0.1% aq TFA), nm (%):405 (100), 501 (14.3), 532 (4.8), 604 (7.2), 663 (39.2); fluorescence λmax. (0.1% aq. TFA, λexc. = 405 nm) 650 nm; HRMS [found (ESI+) 862.8567 [M + 3 H]^3+^, calcd. for C_125_H_195_N_42_O_19_ 862.8522].

### Cell lines and cultivation

Human oral epidermoid carcinoma KB cells obtained from American Type Culture Collection (ATCC) were grown in RPMI medium supplemented with 10% FCS. MC28 cells, a methylcholanthrene-induced rat fibrosarcoma cell line, were grown in DMEM supplemented with 10% FCS at 37 °C in a humidified atmosphere containing 5% CO_2_. Unless otherwise stated materials for the cell studies were purchased from Sigma-Aldrich (Gillingham, UK).

### *In vitro* PDT/PCI phototoxicity studies

KB and MC28 cells were seeded out in 96 well plates overnight. The cells were then incubated separately with either the conjugates, chlorin e_6_ or saporin for 18 h at selected concentrations. Another group of cells were co-incubated with saporin and selected conjugates. Cells were then washed twice with PBS and incubated for a further 4 h with fresh full medium. Irradiation was carried out for up to 5 minutes using a blue LumiSource^®^ flatbed lamp with peak emission at 420 nm and 7 mW cm^−2^ output (PCI Biotech, Oslo, Norway). Cell viability was evaluated at up to 96 h after light illumination using the standard MTT (3-(4,5-dimethylthiazol-2-yl)-2,5-diphenyltetrazolium bromide) assay. Each experiment was carried out in triplicate. Control groups with no drugs added and with or without light were also assessed.

### Cellular uptake and localisation

Cells were seeded in small Petri dishes, with a glass cover slip bottom window for use with an inverted microscope (Fluorodish, World Precision Inst. UK), and allowed to attach overnight. Cells were incubated for 24 h with the compounds (2.5 µM). Afterwards, culture medium was removed and replaced with fresh medium containing Lysotracker Green (100 nM) for 30 min before microscope imaging. Cells were washed 2 times with PBS and incubated with drug-free/phenol red-free medium for confocal imaging using an Olympus Laser scanning confocal microscope (FluoView FV1000, 60x magnification, NA 1.20, Olympus UK Ltd, Essex, UK). Fluorescence from the photosensitiser was recorded within the range of 620–720 nm using a 405 nm laser for the excitation. For LysoTracker Green, cells were illuminated at 488 nm and the fluorescence signal was recorded at 500–550 nm. Colocalisation analysis and image processing of the 16-bit images were performed with ImageJ software.

### Statistical analysis

Data were analysed using the two-tailed Student’s T-test with appropriate testing *post hoc* using Prism 6 software. Error bars from the mean show +/− standard deviation (SD). Values of *P* < 0.05 were considered to be significant. To test for a synergistic interaction between the two separate therapies applied, we used the following equation:

1 $$\propto \,=\,\frac{ \% {V}_{PDT}\,x\, \% {V}_{cytotoxin}}{ \% {V}_{combination}},$$

In the numerator of equation 1, *%V* is the percentage viability for each separate therapy (i.e. PDT and the application of the cytotoxin), and the denominator is the percentage viability observed following the PCI combination treatment^51^. If α > 1 then a synergistic effect has been observed whereas an antagonistic effect is denoted by α < 1. This analysis has been used previously by us and others to identify synergistic effects in PCI^46, 52^.

### Photophysical Characterization

For the singlet oxygen studies, absorption spectra of the compounds were measured using a Perkin-Elmer Lambda 25 UV/Vis spectrometer (Perkin-Elmer, Beaconsfield, UK) with quartz cuvettes. The singlet oxygen phosphorescence at 1270 nm was detected using time-resolved photon counting from aerated solutions in deuterated methanol (CD_3_OD) in quartz cuvettes. For detection in the near-IR, a thermoelectrically cooled photomultiplier (model H10330–45, Hamamatsu Photonics Ltd, Hertfordshire, UK) was used, and the emission was collected via a series of lenses from the cuvette in combination with a long-pass and a band-pass filter centred at 1270 nm (Interferenzoptik Electronik GmbH, Germany). The solutions were excited using a 532 nm Nd:YAG laser (Lumanova GmbH, Hannover, Germany) with the beam axis aligned orthogonally to the collection optics. The laser was pulsed at a repetition rate of 3 kHz and a pulse length of 3 ns, with a mean power output of 8 mW, and a fast photodiode (1 ns rise time, Becker-Hickl, Berlin, Germany) was used to synchronize the laser pulse with the photon counting system. Calibrated neutral density filters were used to attenuate the laser power. The photon counting detection equipment consisted of a multiscaler board (model MSA-300, Becker-Hickl, Berlin, Germany) and a pre-amplifier (Becker-Hickl, Berlin, Germany). Integrated time-resolved phosphorescence traces were analysed using FluoFit software (PicoQuant GmbH, Berlin, Germany) to extract the singlet oxygen decay lifetime. To calculate the quantum yield standard zero-time intercept analysis was used.

## Electronic supplementary material

Supplementary Information
