# Supplementary material for: Endolysosomal targeting of a clinical chlorin photosensitiser for light-triggered delivery of nano-sized medicines
Source: Sci Rep. 2017 Jul 20;7:6059. doi: 10.1038/s41598-017-06109-y (PMC5519633; doi:10.1038/s41598-017-06109-y)
Supplement: Supplementary file 1 — Supplementary Information [file 41598_2017_6109_MOESM1_ESM.pdf]

Electronic Supplementary Material (ESI)

Endolysosomal targeting of a clinical chlorin photosensitiser for light-triggered delivery of nano-sized medicines

Elnaz Yaghini,[a‡*] Ruggero Dondi,[b‡] Kumal Tewari,[b] Marilena Loizidou,[a] Ian M. Eggleston,[b*] and Alexander J. MacRobert,[a*]

[a] Division of Surgery and Interventional Science, University College London, Royal Free Campus, Rowland Hill Street, London NW3 2PE, UK

[b] Department of Pharmacy and Pharmacology, University of Bath, Bath BA2 7AY, UK

[‡] These authors contributed equally.

[*] Corresponding authors: Dr Elnaz Yaghini and Prof. Alexander MacRobert, Division of Surgery and Interventional Science, University College London and Dr Ian Eggleston, Department of Pharmacy and Pharmacology, University of Bath, UK.

Email addresses: I.Eggleston@bath.ac.uk, a.macrobert@ucl.ac.uk, elnaz.yaghini@ucl.ac.uk

FAX number: ++4420-7472 6444

Contents

S2: Chemical Experimental – General Information

Synthesis of 13^1-ethylenediaminylchlorin e_6 dimethyl ester **(1)**

S3: Figure 1: ^{1}H NMR spectrum of **(1)**

S4: Synthesis of 13^1-[4-(3-maleimido)-propanoylamido]ethylamidolchlorin e_6 dimethyl ester **(2)**

Figure 2: ^{1}H NMR spectrum of **(2)**

S5: Figure 3: ^{13}C NMR spectrum, HPLC profile, UV-visible and fluorescence spectra, mass spectrum of **(2)**

S6: Synthesis of 13^1-[(6-dibenzoazacyclooctynecaproateamido)ethylamidochlorin e_6 dimethyl ester **(3)**

S7: Figure 4: ^{1}H NMR and ^{13}C NMR spectra of **(3)**

S8: Figure 5: HPLC profile, UV-visible and fluorescence spectra, mass spectrum of **(3)**

S9: Figure 6: HPLC profile, UV-visible and fluorescence spectra, mass spectrum of conjugate **(7)**

S10: Figure 7: HPLC profile, UV-visible and fluorescence spectra, mass spectrum of conjugate **(8)**

S11: Figure 8: HPLC profile, UV-visible and fluorescence spectra, mass spectrum of conjugate **(9)**

S12: Figure 9: Time-resolved singlet oxygen phosphorescence signal recorded upon excitation of conjugate at 532 nm with increasing NaN_3 concentrations, showing quenching of phosphorescence by azide ions (A: conjugate **7**, B: conjugate **8**); References

Chemical Experimental - General Information

Chemical reagents were purchased from Sigma-Aldrich, Fluka, Acros, Novabiochem, and Bachem. Peptide grade DMF was purchased from Rathburn Chemicals. Anhydrous DCM was obtained by distillation over calcium hydride. Analytical TLC was performed using silica gel 60 F_{254} pre-coated on aluminium sheets (Merck). Column chromatography was performed on silica gel 60 (35-70 micron) from Sigma-Aldrich. UV spectra were recorded on a Perkin-Elmer Lambda 19 uv/vis spectrophotometer. Fluorescence spectra were recorded on a Cary Eclipse fluorimeter. ^{1}H and ^{13}C NMR were recorded using a Varian Mercury-VX spectrometer at 400 MHz (^{1}H) and 100 MHz (^{13}C) or a Bruker Avance III 500 at 500 MHz (^{1}H) and 125 MHz (^{13}C). Chemical shift values are given in ppm (δ). J values are given in Hz. Analytical RP-HPLC was performed on a Dionex Ultimate 3000 system (Dionex, UK), with a VWD-3400 variable wavelength detector, and a RF-2000 fluorescence detector. Analyses were performed at 35 ± 0.1 °C on a Gemini 5 µ C18 110A column, (150 x 4.6 mm - Phenomenex, UK), equipped with a Security Guard C18 (ODS) 4 x 3.0 mm ID guard column (Phenomenex, UK), at a flow rate of 1 mL/min. Mobile phase A was 0.1% aq. TFA, mobile phase B was 0.1% TFA in MeCN. (Gradient: 0.0–10.0 min 0-95% B, 10.0–20.0 min 95% B, 20.0–20.1 min at 95-5% B, 20.1–23.0 min 5% B). Preparative RP-HPLC was performed on a Dionex HPLC system equipped with a Phenomenex Gemini 5 µ C18 (250 x 10w mm) column at a flow rate of 2.5 mL/min. High resolution mass spectrometry was performed using a Bruker MicroTOF autospec ESI mass spectrometer.

13^{1}-Ethylenediaminylchlorin e_6 dimethyl ester (1)

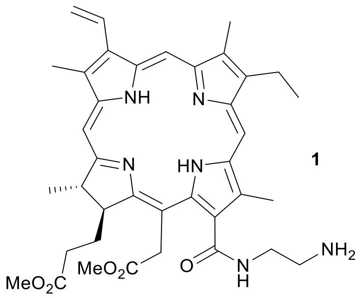

1

Methyl pheophorbide a was prepared by extraction and purification of pheophytin a from *Spirulina spp.* followed by methanolysis of the phytol group.[1] A solution of pheophorbide a (100 mg, 0.17 mmol) in anhydrous THF (25 mL) was treated with 1,2-diaminoethane (397 µL, 5.94 mmol, 36 eq). The resulting solution was stirred at room temperature overnight. The mixture was then diluted with DCM (100 mL) and washed with H_2O (3 x 50 mL) and brine (50 mL). The organic phase was dried (Na_2SO_4) and the solvent was evaporated. The crude product (107 mg, quant.) was used without further purification. A sample was purified by column chromatography, eluting first with 2.5% MeOH/DCM, then 50% MeOH/DCM. The spectroscopic data (^{1}H NMR) was consistent with the literature.[2] ^{1}H-NMR (400 MHz, $(CD_3)_2CO$) 9.86 (s, 1H), 9.79 (s, 1H), 9.12 (s, 1H),

8.29 (dd, *J* = 17.9, 11.6, 1H), 6.46 (dd, *J* = 11.6, 1.5, 1H), 6.19 (dd, *J* = 11.6, 1.5, 1H), 5.64 (d, *J* = 19.0, 1H), 5.41 (d, *J* = 19.0, 1H), 4.68 (q, *J* = 7.2, 1H), 4.54 (q, *J* = 10.1, 1H), 4.19-4.11 (m, 1H), 4.06-3.97 (m, 2H), 3.87 (q, *J* = 7.6, 2H), 3.80-3.50 (m, 14H), 3.36 (s, 3H), 2.77-2.66 (m, obsc, 1H), 2.39-2.30 (m, 4H), 1.78-1.71 (m, 6H), -1.53 (s, 0.5H), -1.84 (s, 0.5H)

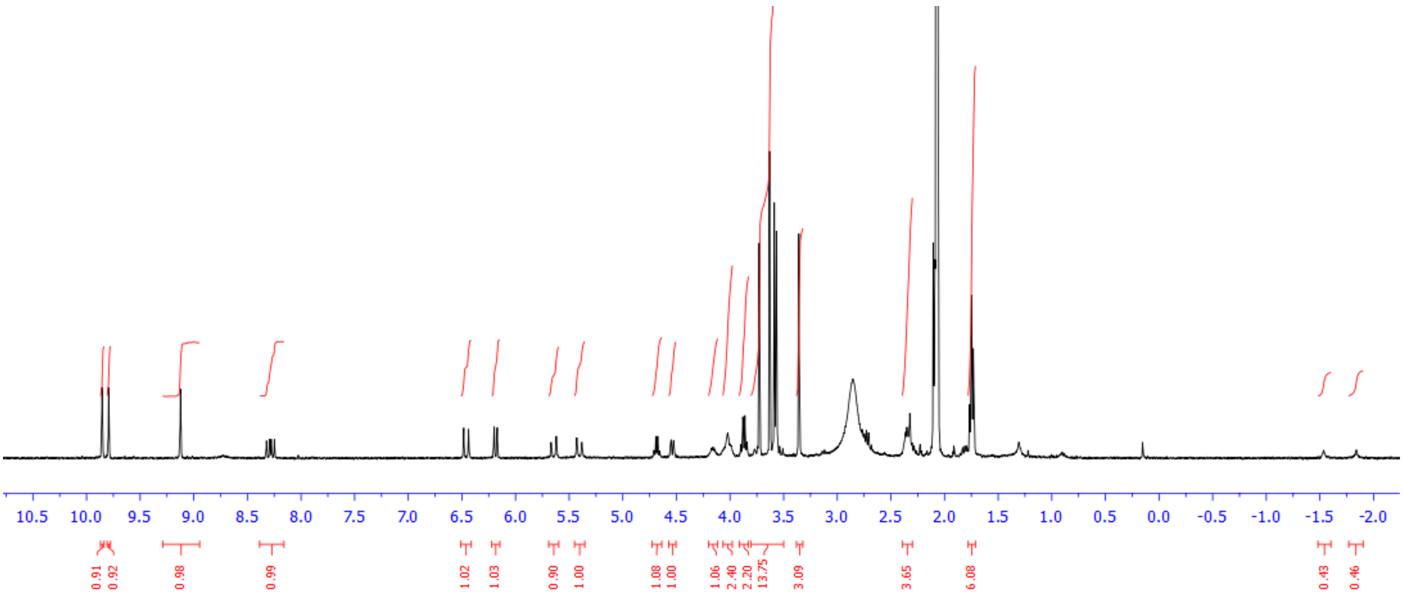

Figure 1. ^{1}H NMR in $(CD_3)_2CO$ of **1**.

13[1]-[4-(3-maleimido)-propanoylamido]ethylamidochlorin e_6 dimethyl ester (2)

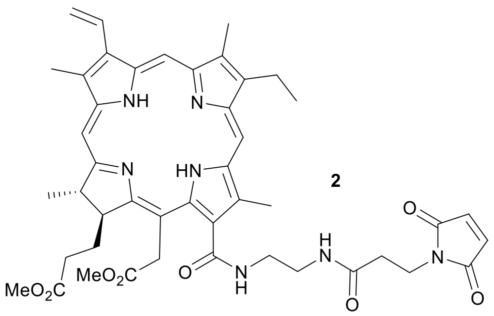

2

A solution of **1** (444 mg, 0.666 mmol) in dry DCM (30 mL) was treated with 3-maleimidopropanoic acid (124 mg, 0.733 mmol), EDC (153 mg, 0.799 mmol), HOBt (108 mg, 0.799 mmol) and DIPEA (346 μL, 2.00 mmol). The mixture was stirred at room temperature overnight, then it was diluted with DCM (100 mL) and was washed with H_2O (100 mL) and brine (100 mL). The organic phase was dried (Na_2SO_4), filtered, and the solvent was evaporated. The crude product was purified by column chromatography (MeOH/EtOAc gradient). This gave **2** as a dark green solid (422 mg, 80%) R_f (EtOAc) = 0.17; R_t HPLC: 8.97 min; UV-vis ($CHCl_3$), nm (%): 404 (100), 511 (8.0), 534 (5.2), 610 (5.9), 665 (32.7); fluorescence $\lambda_{max.}$ ($CHCl_3$, $\lambda_{exc.}$ = 405 nm) 671 nm; ^{1}H-NMR (400 MHz, $CDCl_3$) 9.69 (s, 1H), 9.57 (s, 1H), 8.85 (s, 1H), 7.98 (t, *J* = 14.5, 1H), 6.87 (br, 1H), 6.27 (d, *J* = 17.9, 1H), 6.09 (d, *J* = 11.5, 1H), 5.95 (s, 2H), 5.49 (d, *J* = 18.4, 1H), 5.18 (d, *J* = 18.7, 1H), 4.49 (d, *J* = 7.3, 1H), 4.41 (d, *J* = 9.7, 1H), 3.79-3.55 (m, 9H), 3.27-3.51 (m, 6H), 3.14-3.21 (m, 2H), 2.62-2.50 (m, 1H), 2.13-2.36 (m, 3H), 1.81-1.51 (m, 7H), 1.42-1.16 (m, 6H), 0.94-0.76 (m, 2H), -1.83 (br, 1H), -1.95 (br, 2H); ^{13}C-NMR (100 MHz, $CDCl_3$) 174.2, 173.6, 170.8, 170.4, 169.9, 134.9, 133.8, 130.7, 129.3, 122.3, 101.4, 98.8, 94.3, 75.7, 53.2, 52.4, 51.8, 49.3, 40.1, 39.7, 38.0, 34.8, 34.3, 32.0, 31.5, 31.2, 30.4, 30.3, 29.8, 29.5, 27.8, 27.2, 26.3, 23.2, 22.8, 19.7, 17.7, 14.2, 12.2, 12.1, 11.4; HRMS [found (ESI+) 818.3875 $[M+H]^+$], calcd. for $C_{45}H_{52}N_7O_8$: 818.3872].

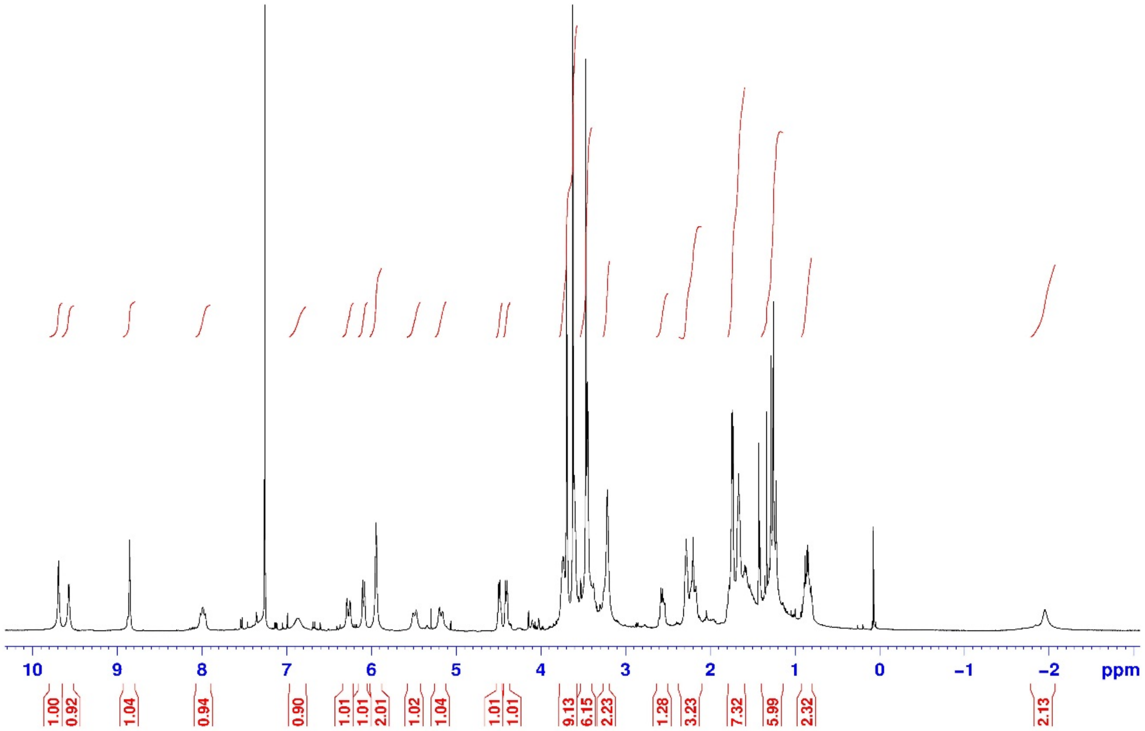

Figure 2. ^{1}H NMR in $CDCl_3$ of **2**.

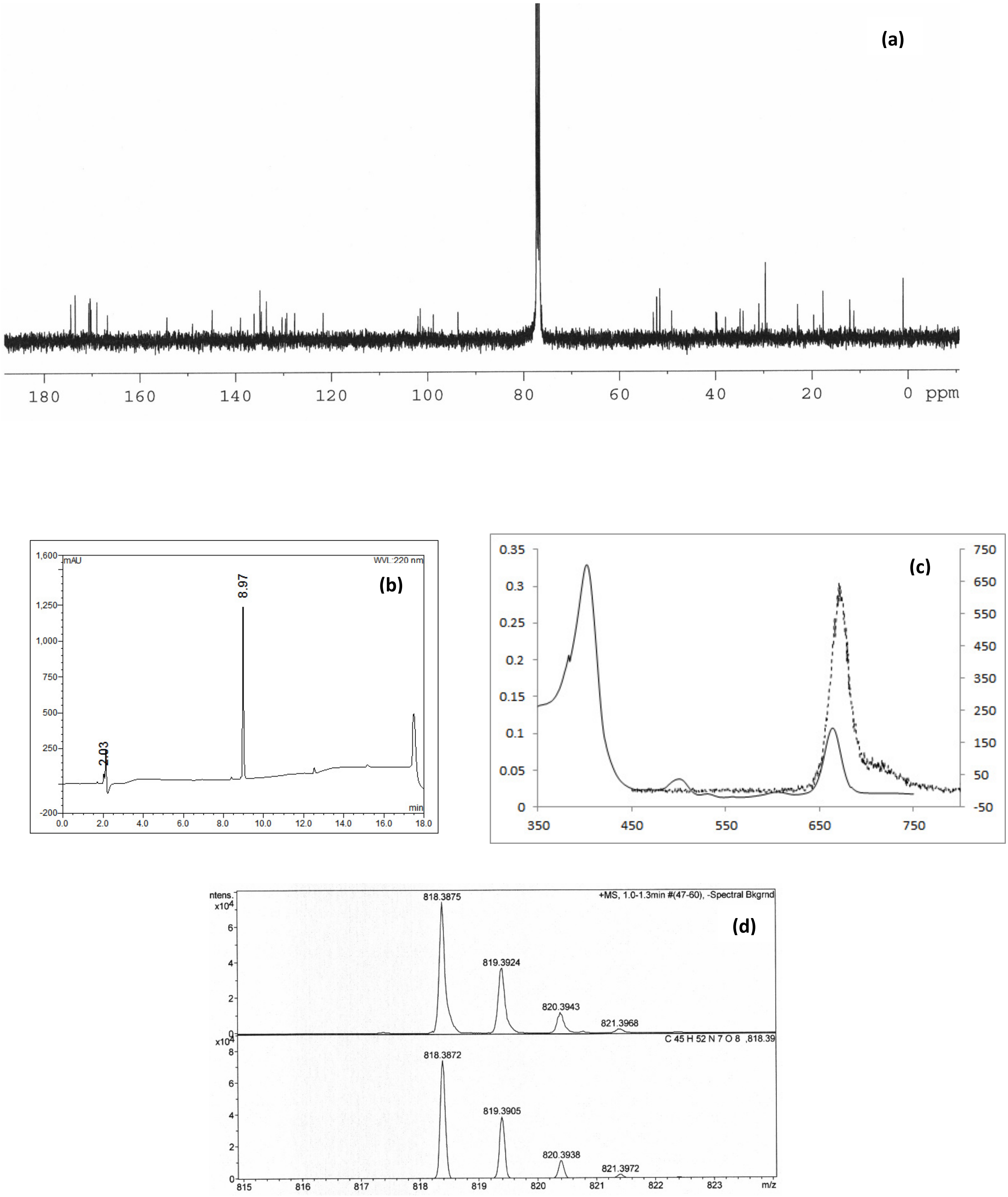

Figure 3 (a) ^{13}C NMR in $CDCl_3$ of **2**; **(b)** HPLC profile of **2**; **(c)** UV-visible (solid line) and fluorescence (dotted line) spectra in $CHCl_3$ for **2**, **(d)** ESI-MS for **2**.

13^1-[(6-dibenzoazacyclooctynecaproateamido)ethylamidochlorin e_6 dimethyl ester (3)

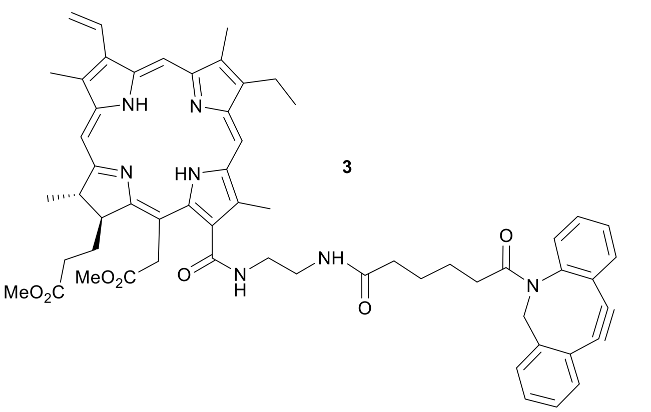

A solution of **1** (22.0 mg, 0.033 mmol) in dry DCM (1 mL) was treated with HOBt (4.9 mg, 0.036 mmol), dibenzocyclooctyne-acid (10.0 mg, 0.030 mmol), EDC.HCl (6.9 mg, 0.036 mmol) and DIPEA (15.6 µL, 0.09 mmol). in anhydrous DCM (1 mL). The mixture was stirred at room temperature overnight, then the solvent was evaporated and the crude product was purified directly by column chromatography, eluting with EtOAc. This gave **3** as a green solid (13 mg, 44 %); R_f (EtOAc) = 0.23; R_t (HPLC) = 10.36 min; UV-vis: λ_{max} nm (%): 404 (100), 489 (10.0), 609 (4.2), 665 (32.9); fluorescence $\lambda_{max.}$ ($\lambda_{exc.}$ = 420 nm) 667 nm; ^{1}H-NMR (500 MHz, DMSO-d_6, 60°C) 9.83 (br, 1 H), 9.78 (br, 1 H), 9.13 (br, 1 H), 8.94 (br, 1 H), 8.29 (dd, *J* = 17.8, 11.6, 1 H), 7.73 (br, 1 H), 7.66 - 7.25 (m, 8 H), 6.46 (d, *J* = 17.8, 1 H), 6.21 (d, *J* = 11.6, 1 H), 5.53 (d, *J* = 18.8, 1 H), 5.31 (d, *J* = 18.8 Hz, 1 H), 5.10 - 5.01 (m, 1 H), 4.63 (d, *J* = 7.1 Hz, 1 H), 4.45 (d, *J* = 10.4, 1 H), 3.90-3.81 (m, 2 H), 3.73-3.65 (m, 4 H), 3.63-3.43 (m, 12 H), 3.38 - 3.29 (m, 3 H), 2.74 - 2.60 (m, 1 H), 2.37 - 2.26 (m, 1 H), 2.24-2.11 (m, 2 H), 2.04 - 1.94 (m, 2 H), 1.88 - 1.76 (m, 1 H), 1.73 - 1.60 (m, 7 H), 1.44 - 1.18 (m, 6 H), -2.15 - -1.70 (m, 2 H); ^{13}C-NMR (125 MHz, DMSO-d_6, 60°C) 174.3, 173.5, 173.1, 172.6, 172.1, 168.6, 168.4, 152.4, 148.9, 144.6, 138.9, 136.3, 134.4, 132.8, 130.8, 130.5, 130.2, 129.8, 129.2, 128.5, 128.4, 128.0, 127.2, 125.5, 123.0, 122.6, 122.0, 114.9, 108.6, 103.6, 101.2, 98.9, 94.9, 79.7, 79.4, 79.2, 55.3, 53.3, 52.2, 51.7, 48.7, 38.8, 37.5, 35.7, 34.4, 34.2, 33.8, 31.1, 30.0, 25.1, 25.0, 24.8, 24.3, 23.4, 19.4, 18.0, 12.4, 12.1, 11.4; HRMS [found (ESI+): 982.4995 $[M+H]^+$, calcd. for $C_{59}H_{63}N_7O_7$: 982.4862].

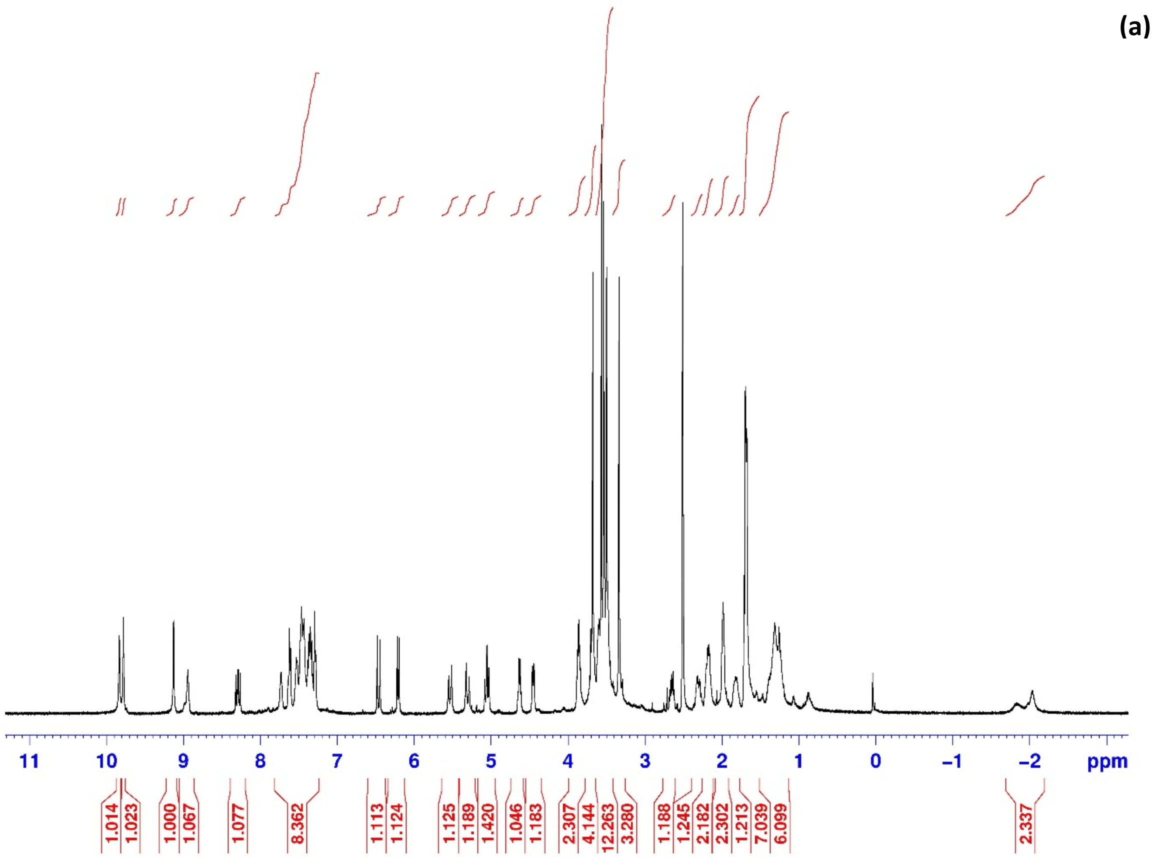

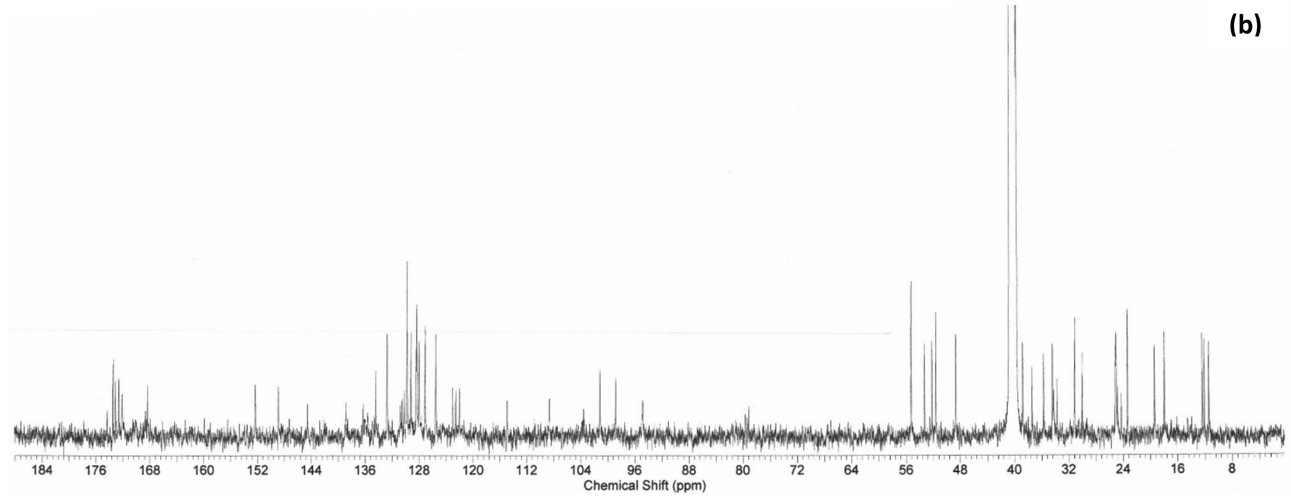

Figure 4 (a) ^{1}H NMR and **(b)** ^{13}C NMR in DMSO-d_6 of **3**.

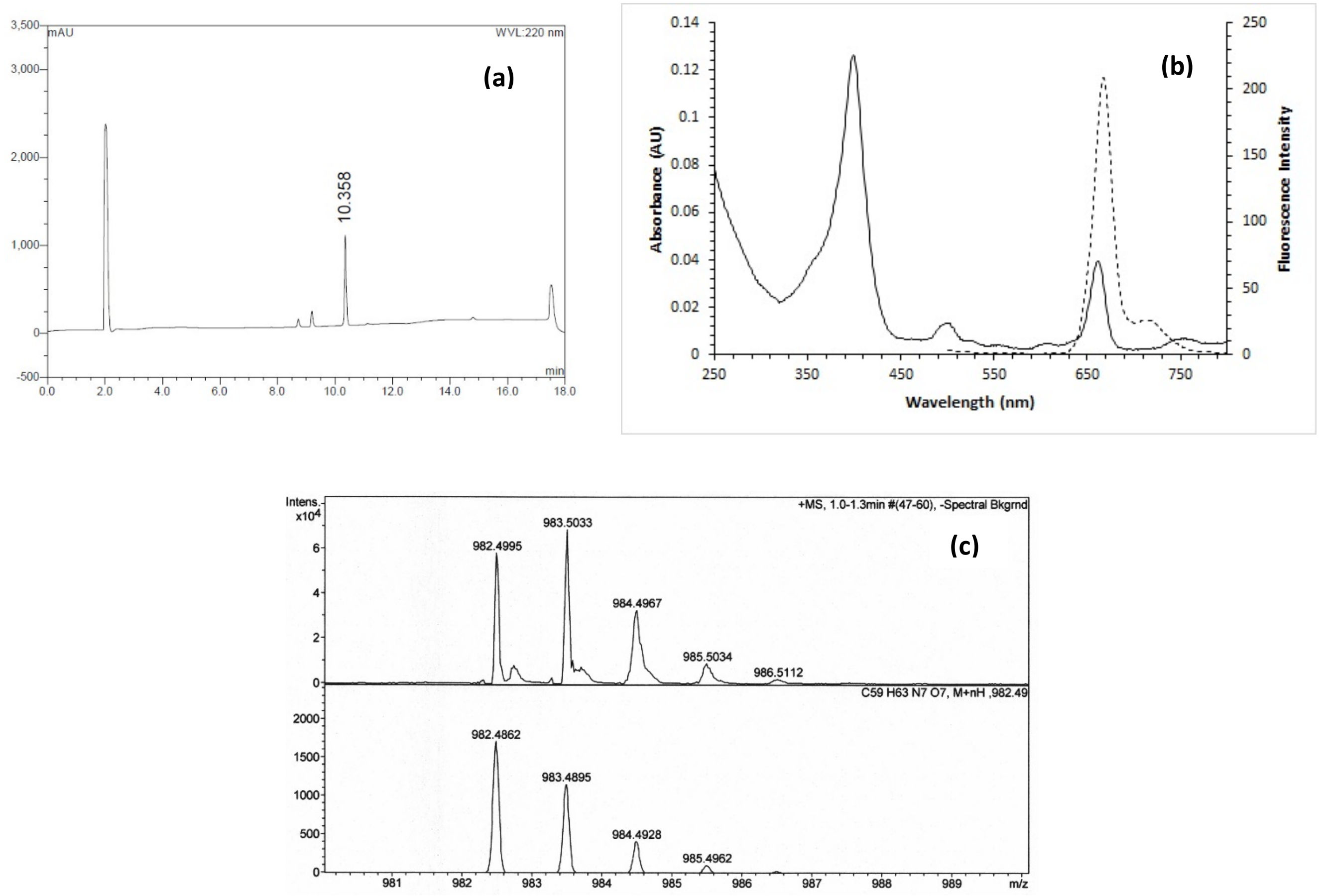

Figure 5 (a) HPLC profile of **3**; **(b)** UV-visible (solid line) and fluorescence (dotted line) spectra in $CHCl_3$ for **3**; **(c)** ESI-MS for **3**.

Ce_6-Tat conjugate (7) – *N-terminal thiol maleimide ligation* (Ce_6 refers to chlorin e_6)

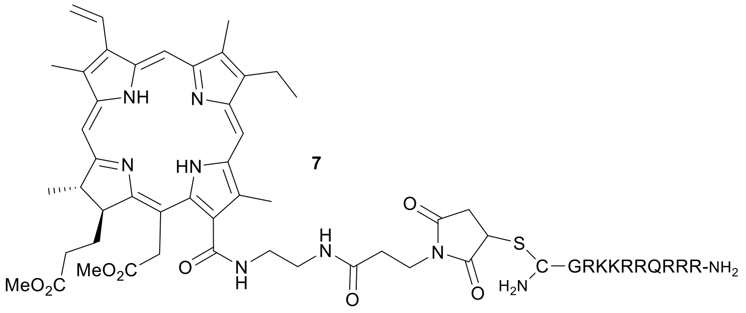

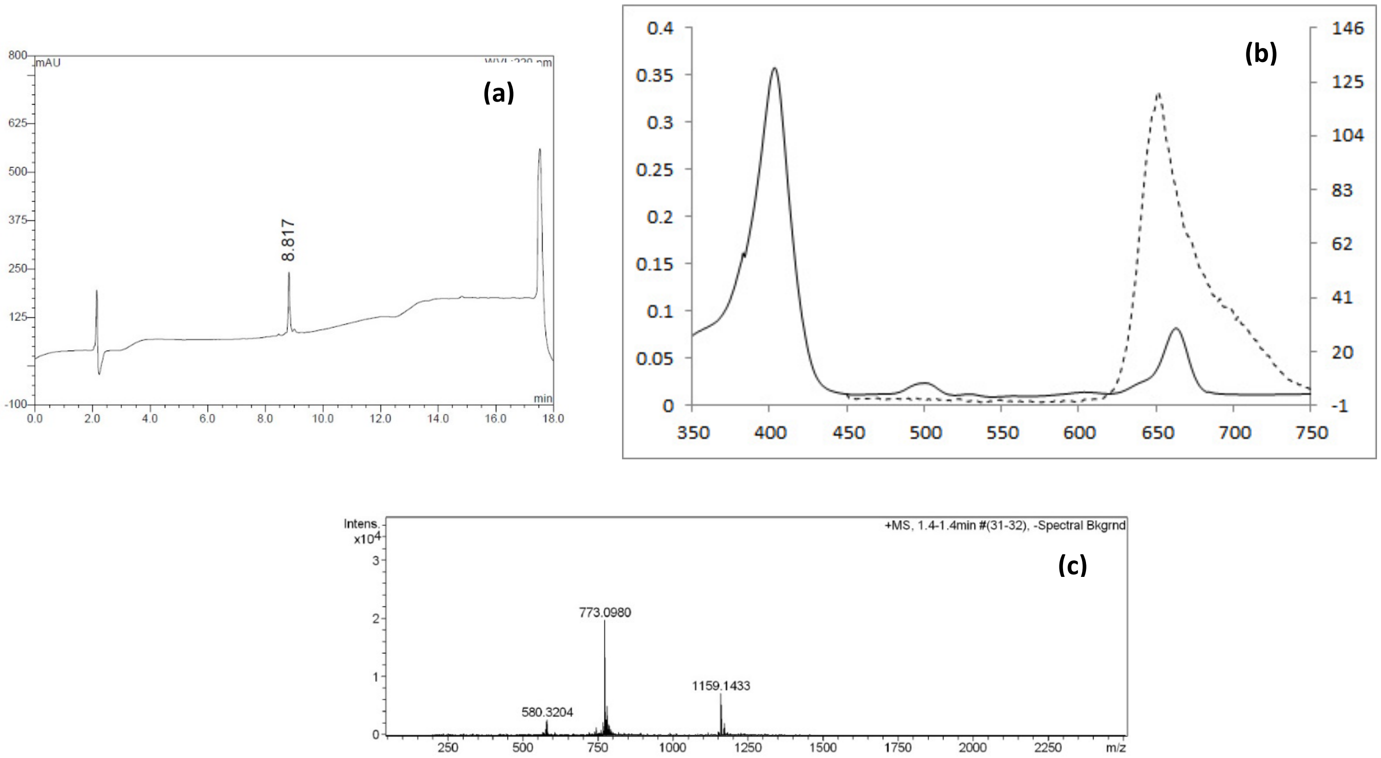

Figure 6 (a) HPLC profile of **7**; **(b)** UV-visible (solid line) and fluorescence (dotted line) spectra in 0.1% aq. TFA for **7**; **(c)** ESI-MS for **7**.

Ce$_6$-Tat conjugate (8) – *C-terminal thiol maleimide ligation*

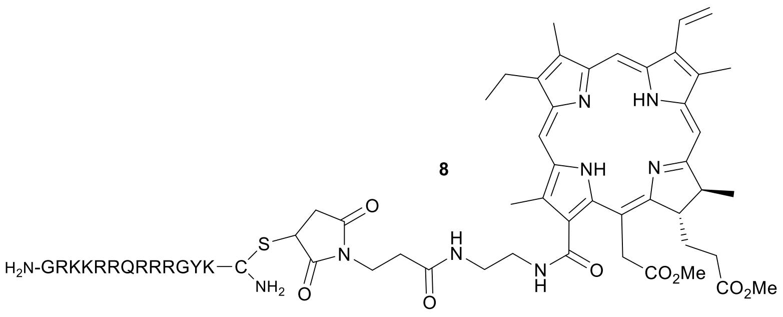

8

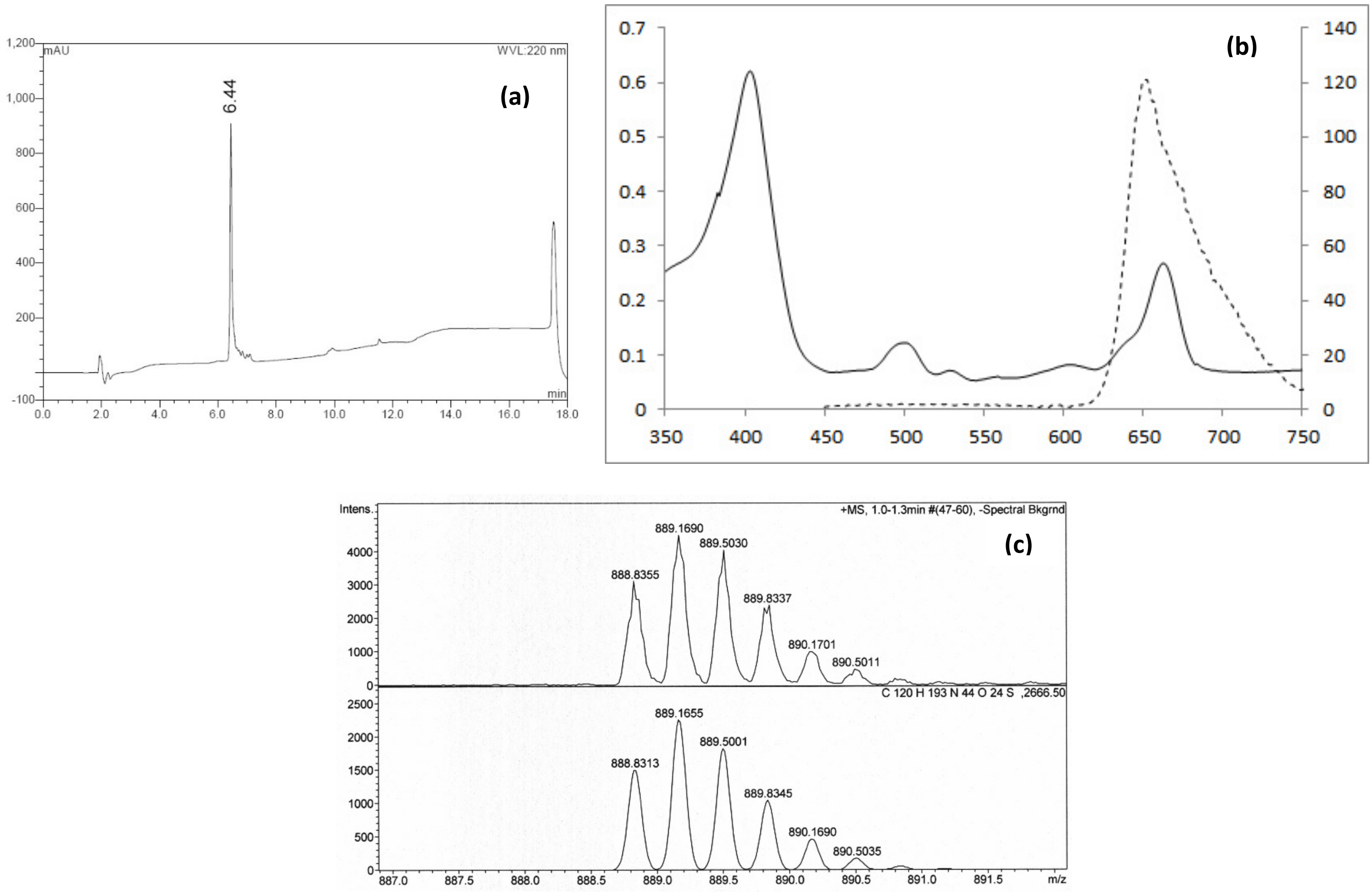

Figure 7 (a) HPLC profile of **8**; **(b)** UV-visible (solid line) and fluorescence (dotted line) spectra in 0.1% aq. TFA for **8**; **(c)** ESI-MS for **8**.

Ce6-Tat conjugate (9) – *N-terminal SPAAC ligation*

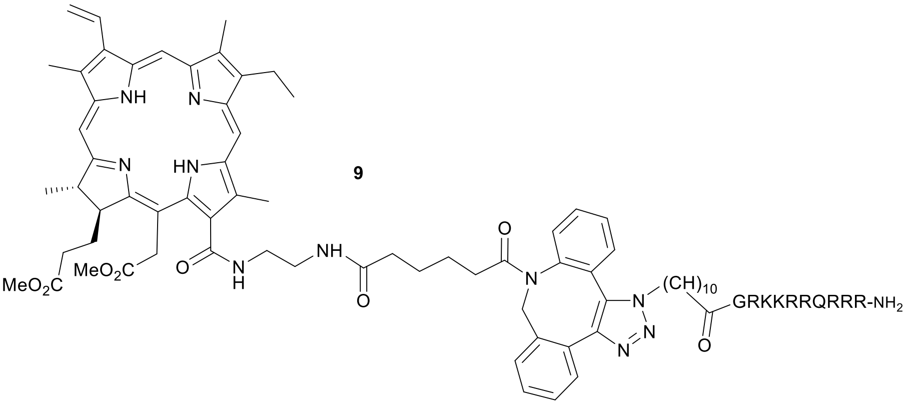

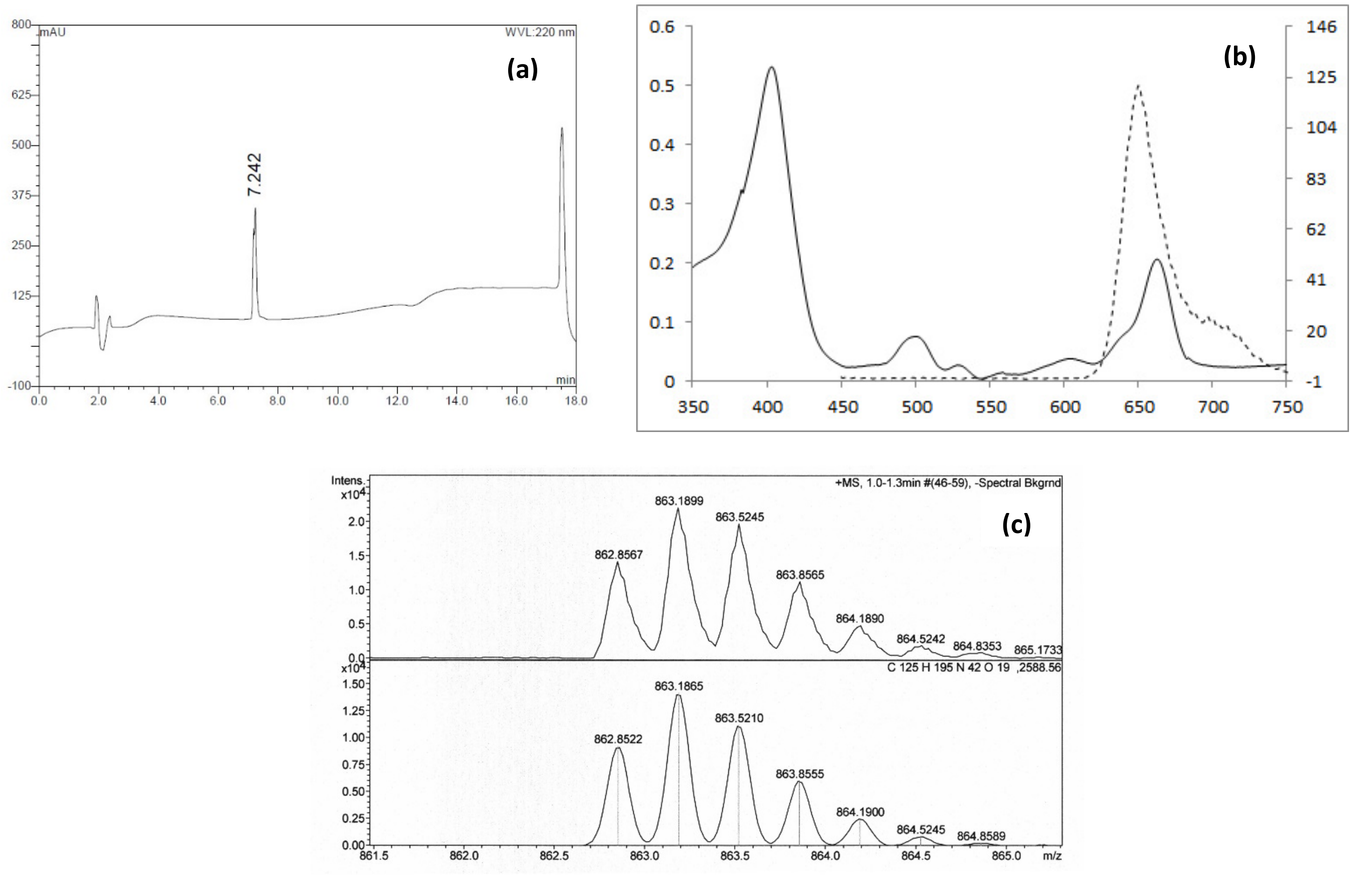

Figure 8 (a) HPLC profile of **9**; **(b)** UV-visible (solid line) and fluorescence (dotted line) spectra in 0.1% aq. TFA for **9**; **(c)** ESI-MS for **9**.

Singlet oxygen quenching using sodium azide

To confirm that the detected singlet oxygen ($^1O_2$) phosphorescence curve was due to the decay curve of $^1O_2$, different concentrations of NaN_3 were added to solutions of chlorin e_6 conjugates (**7** and **8**), dissolved in deuterated methanol and the 1270 nm decay kinetics for each sample solution was measured. As shown in Figure 8, the $^1O_2$ signal was strongly quenched, on addition of NaN_3, consistent with the detected signal being due to singlet oxygen. This is consistent with the rapid physical quenching of singlet oxygen by azide ions.[3]

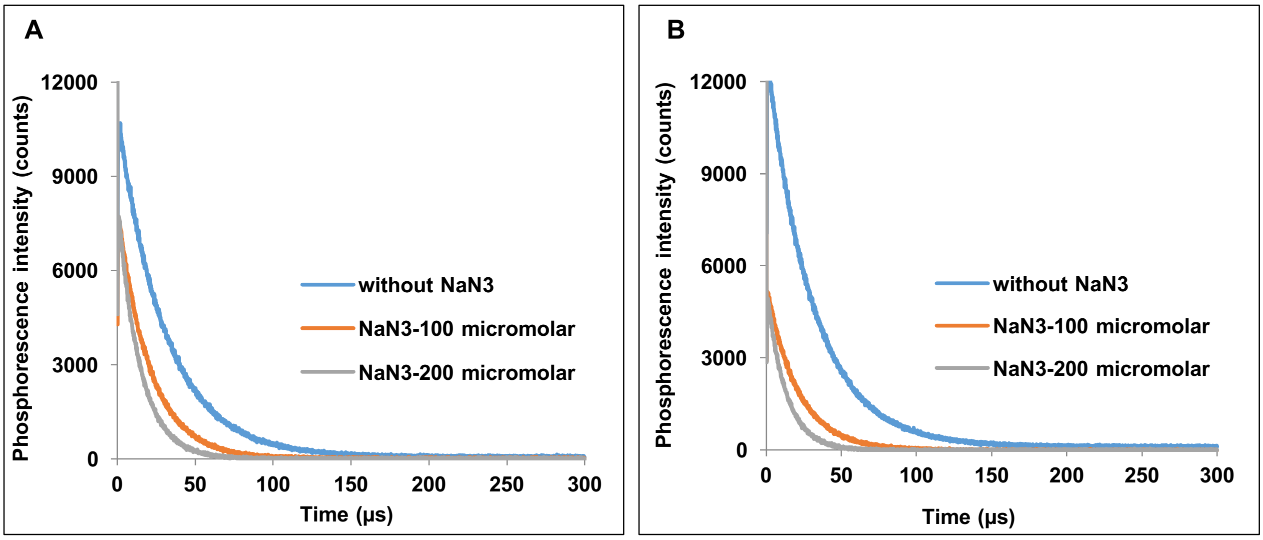

Figure 9 Time-resolved singlet oxygen phosphorescence signal recorded upon excitation of conjugate at 532 nm with increasing NaN_3 concentrations, showing quenching of phosphorescence by azide ions (A: conjugate **7**, B: conjugate **8**)

Reference List

(1) Smith, K.M.; Goff, D.A.; Simpson, D.J. The meso substitution of chlorophyll derivatives: direct route for transformation of bacteriopheophorbides d into bacteriopheophorbides c. *J Am Chem Soc* **1985**, 107, 4946-4954.

(2) Jinadasa, R.G.; Hu, X.; Vicente, MG.; Smith, K.M. Syntheses and cellular investigations of 17(3)-, 15(2)-, and 13(1)-amino acid derivatives of chlorin e(6). *J Med Chem* **2011**, 54, 7464-7476.

(3) Li ,M.Y.; Cline, C.S.; Koker, E.B.; Carmichael, H.H.; Chignell, C.F.; Bilski, P. Quenching of singlet molecular oxygen (1O2) by azide anion in solvent mixtures. *Photochem Photobiol* **2001**, 74, 760-764.
